# Transient feedforward communication between somatosensory and premotor cortex supports goal-directed behavior

**DOI:** 10.1371/journal.pbio.3004095

**Published:** 2026-10-29

**Authors:** Yadollah Ranjbar-Slamloo, Tsukasa Kamigaki

**Affiliations:** Lee Kong Chian School of Medicine, Nanyang Technological University, Singapore, Singapore; Institute of Science and Technology Austria, AUSTRIA

## Abstract

Goal-directed behavior requires sensory information to be transformed into motor commands, yet the precise temporal structure of communication between sensory and motor cortices during this process remains poorly understood. By simultaneously recording spiking activity from primary somatosensory cortex (S1) and secondary motor cortex (M2) in mice performing a tactile delayed go/no-go task, we identified a brief, stimulus-triggered communication window that predicted behavioral success. Time-shifted canonical correlation analysis revealed a sharp, feedforward surge in S1 → M2 coupling peaking at a short latency, as expected for a potentially direct transmission pathway. This transient interaction conveyed stimulus-selective information such that the S1 → M2 communication subspace aligned with stimulus-coding axes in both regions. In contrast, M2 → S1 feedback interactions were weaker and delayed. Brief optogenetic suppression of S1 reduced both M2 stimulus selectivity and behavioral discriminability, consistent with a functional role of the transient S1 → M2 coupling. These findings demonstrate that sensorimotor transformation is supported not by sustained linear coupling along the stimulus- or choice- axis, but by a short-lived and highly selective feedforward communication channel that rapidly transmits reliable sensory representations into motor-planning circuits.

## Abstract 2

Goal-directed behavior requires sensory information to be transformed into motor commands, but the structure of this sensorimotor communication in the brain is unclear. This study shows that brief, selective, feedforward coupling transmits sensory representations into motor-planning circuits.

## Introduction

The brain constantly receives sensory input from peripheral sense organs and one of its most crucial functions is to route relevant information along the sensorimotor axis to generate appropriate action. We have a strong foundation of knowledge about how local circuits shape sensory representations [1–3], which circuit elements maintain task-relevant information in frontal cortical regions [4–9], and which brain regions are necessary for sensorimotor transformations during goal-directed perceptual behavior [10–15]. Extensive anatomical connectivity maps further provide a structural scaffold for interpreting these functional processes. However, critical gaps persist and occlude our understanding of sensorimotor transformation. When, where, and how sensory information is transformed into motor actions are yet to be clarified.

The rodent whisker system has served as a powerful model for studying tactile sensorimotor transformations [16–20]. Tactile input first arrives in the barrel-field area of primary somatosensory cortex (S1), and is transformed into lick responses through downstream areas, including secondary somatosensory cortex, premotor regions of the frontal cortex, and primary motor area [12,15,19,21,22]. Additional areas may be involved depending on the complexity of behavioral tasks [10]. Specifically, medial parts of the prefrontal cortex respond to sensory input in a conditional manner, which emerges with learning and disappears when the sensory input is no longer rewarding [15,23]. Recordings and perturbations across these nodes of the sensory pathway have revealed the signal content of each region and their necessity for perceptual decisions [11,12]. However, a full understanding of sensorimotor transformations requires not only a catalogue of regional signals but also a mechanistic account of how task-relevant information is transmitted between distant cortical areas.

Interactions between distant brain regions have been studied using various physiological techniques [24–28] and computational approaches [29–31]. Many studies have relied on coherence measures between local field potentials or between spikes and LFPs, which can assess large-scale interareal synchronization [25,27,28,32–34] but offer limited insight into the underlying cellular-resolution mechanisms. Recent advances in recording techniques now allow simultaneous measurement of spiking activity from large numbers of neurons across multiple regions during well-defined tasks [35–37]. These developments have in turn necessitated population-level analyses, which move beyond single-cell measures to capture how distributed neuronal ensembles interact [38–42]. These approaches can reveal how ensembles of single neurons in one region relate to those in another, thereby identifying what information is transferred, in which direction, and with what precise time during task performance [39,43–48].

Reciprocal synaptic connections between somatosensory and frontal regions [49–51] support a putative feedforward flow of sensory information and a feedback flow of memory-, choice-, motor- and behavioral state-related signals [19,21,22,45]. However, during behavior, moment-to-moment interactions can be confounded by ongoing sensory input, internal state fluctuations, and convergent signals from other regions. Coherence-based approaches, which average over broad time windows and aggregate signals across large neural populations, cannot separate these distinct sources of covariance. As a result, apparent interareal synchrony may reflect shared modulations rather than genuine task-related sensorimotor transformations. Disentangling background activity from task-relevant communication between sensory and premotor regions therefore requires an analytical framework with sufficient temporal precision to capture fast interareal dynamics [39] and, in parallel, to relate these dynamics to sensory-coding [44] or decision-related activity patterns.

To address this, we simultaneously recorded spiking activity in S1 and M2 in a go/no-go task requiring discrimination of tactile stimuli. Using a time-shifted canonical correlation analysis (CCA) with high temporal resolution, we revealed a transient S1–M2 interaction shortly after stimulus presentation, followed by a rapid decoupling of the two regions. The S1–M2 communication subspace was aligned with stimulus-coding subspace within each region, and its strength predicted behavioral performance, demonstrating that the communication channel carried task-relevant sensory information. Furthermore, brief optogenetic suppression of S1 during stimulus presentation demonstrated that S1 activity is required for both M2 stimulus selectivity and successful task performance, consistent with a functional role of the rapid feedforward interaction. Together, our findings uncover a short-lived communication channel between S1 and M2 that supports the conversion of sensory information into motor responses.

## Results

### Behavioral task and dual-site recordings

We investigated interactions between primary somatosensory cortex (S1) and secondary motor cortex (M2) by simultaneously recording neuronal activity in mice performing a tactile delayed go/no-go task (Fig 1A–1E). In this task, a brief air puff was delivered unilaterally to the whiskers, followed by a 1-s delay, a 2-s auditory-cued response window, and a 5.1–7.0-s inter-trial interval. In go trials, a left-sided air puff prompted mice to extend their tongue, break an IR beam, and receive a water reward (Fig 1A and 1B). No-go trials had the same events and timing as go trials, except that the air puff was delivered to the opposite side and mice were required to withhold licking (Fig 1B and 1C). Mice were trained to stable performance before recordings (S1A–S1C Fig) and typically achieved d-prime (*d*′) >1.5 during electrophysiological sessions (S1D Fig).

**Fig 1:**
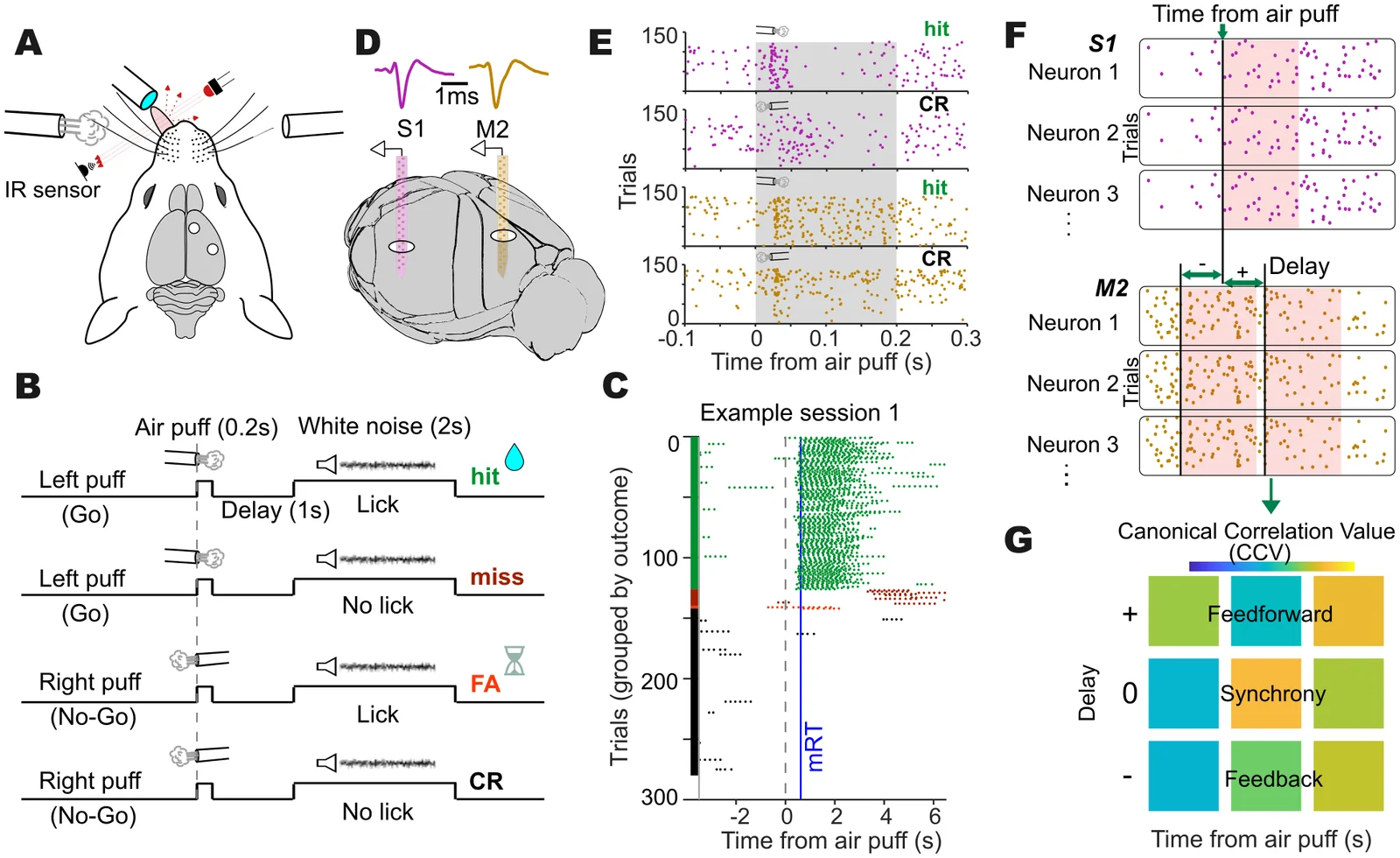
Experimental design and data analysis approach. **(A)** Schematic top view of the mouse head in the delayed go/no-go task (mouse head, modified from reference [52]). **(B)** Trial types and timing of task events. **(C)** Lick raster of an example recording session grouped by trial outcomes. Green: hit, dark red: miss, light red: false alarm (FA), black: correct rejection (CR). mRT: median reaction time. **(D)** Dual-site recording from primary somatosensory (S1) and secondary motor (M2) cortices during the task. Purple: spike waveform of an example neuron in S1, orange: example neuron recorded from M2. **(E)** Raster plot of the example neurons in hit and CR trials, color-coded for each region (purple: S1, orange: M2). Shaded regions indicate air puff duration (0.2 **s)**. **(F)** Schematic description of key variables in the time-shifted canonical correlation analysis. Rectangular panels represent neurons and dots represent spikes within each trial. Vertical red shades represent the time window (50 ms) used for CCA computation with 5-ms binning of spikes. Horizontal green arrows depict temporally delayed time windows in M2. **(G)** Schema showing output of the analysis as one canonical correlation value (CCV, color in rectangles) for each time window and delay relative to the air puff. The sign of delay can inform whether the communication is feedforward or feedback, and the absolute value of delay informs the level of synchrony.

Recordings targeted S1 and M2 subregions within the whisker sensorimotor subnetwork [49] (Figs 1D, S1E and S1F). As shown in two example sessions, both regions exhibited strong initial responses to contralateral stimuli and weaker responses to ipsilateral stimuli (Figs 1E and S2A–S2H). In S1, the contralateral stimulus (both hit and miss trials) evoked reliable initial responses within 50 ms of stimulus onset in many neurons [53–55] (S2A, S2B, S2E and S2F Fig). In M2, population activity also showed strong initial responses to contralateral stimuli (hit trials), whereas responses to ipsilateral stimuli (CR trials) were weaker (S2C, S2D, S2G and S2H Fig). To quantify stimulus preference, we calculated a selectivity index for each neuron using the first 100-ms time window following stimulus onset. In this time window, M2 populations had a much narrower range of selectivity than S1 (S3A Fig). Across 50 recording sessions, the median fraction of S1 neurons with significant selectivity within 100 ms of stimulus onset was 0.29. We used this value to classify sessions into high- or low-selectivity (S3B–S3G Fig), reflecting whether the air puff effectively engaged the S1 receptive fields.

### Transient S1–M2 communication predicts behavioral performance

We employed time-shifted CCA [43] to characterize population-level communication between S1 and M2 (Fig 1F and 1G). This analysis identifies feedforward and feedback interactions as well as any task-specific synchronizations between the two regions [43,56]. To understand overall interactions, including shared tuning properties between the two regions, we first computed total correlation, which preserves all possible interactions, and compared it with “noise correlation,” which reflects trial-to-trial co-fluctuations in activity between S1 and M2 by subtracting each neuron’s trial-averaged activity [41,43,48]. We first examined correlation maps of the two example sessions introduced earlier (in S2 Fig). Total correlation revealed focal, synchronous interactions shortly after the stimulus (Fig 2A and 2B, top left). Enhanced, transient interactions were also observable when go (left air puff) and no-go (right air puff) trials were separately analyzed (Fig 2A and 2B, middle and bottom left). In noise correlation, we observed much weaker stimulus-locked interactions compared to the total correlation (Fig 2A and 2B, right).

**Fig 2:**
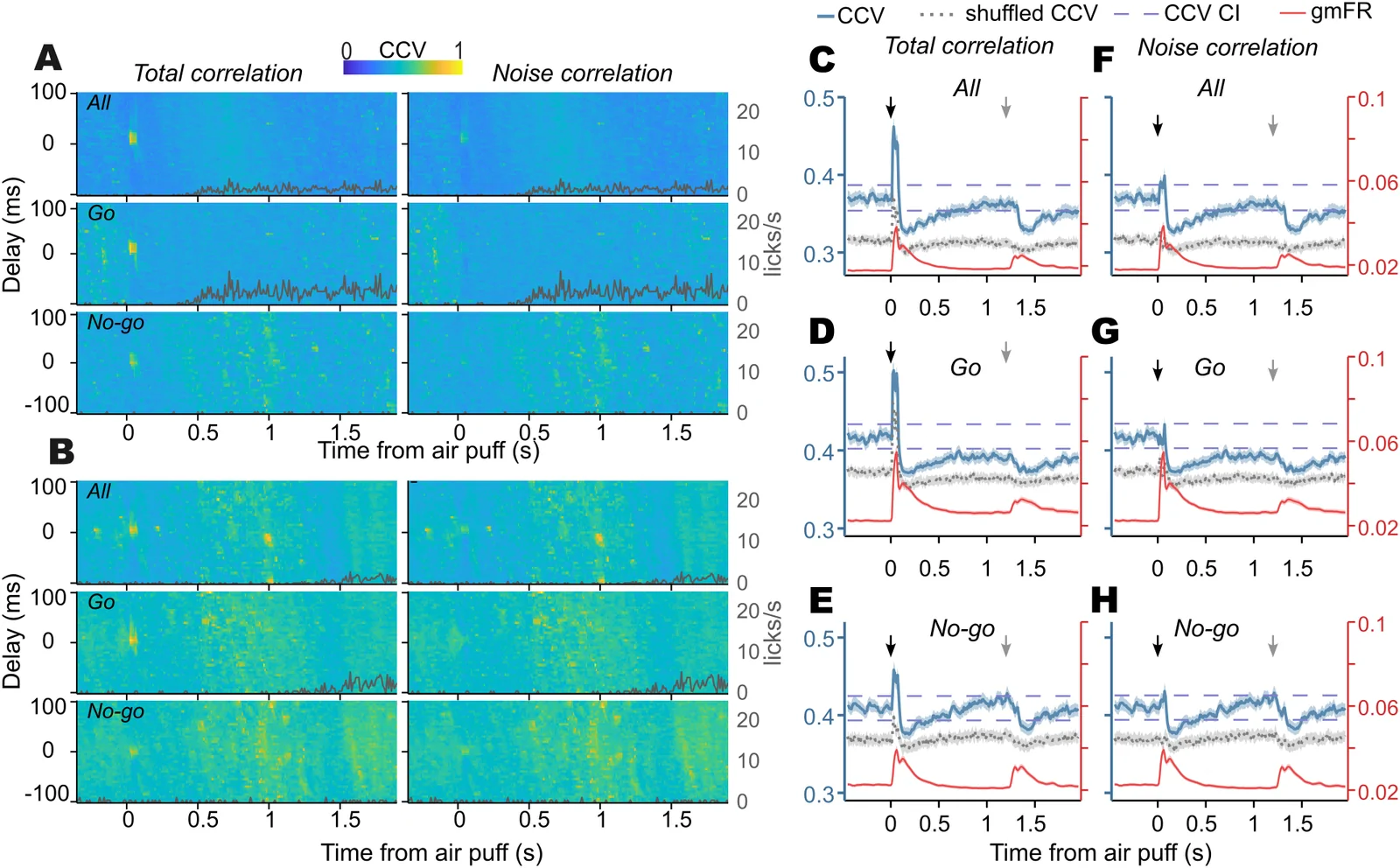
Canonical correlation analysis reveals a transient interaction during the air puff. **(A and B)** Canonical correlation values (CCV) of the two example sessions (Example 1 and Example 2, respectively, as in S2 Fig), showing total correlation (left) and response-subtracted interactions or noise correlation (right) between S1 and M2 for all (top), go (middle), and no-go trials (bottom). Lick responses are plotted in gray on the right y-axis. **(C–E)** Average CCV across ±10 ms delays and across 50 sessions. CCV in all trials (C, *p*_up_ = 1.9 × 10^−7^, *z*_up_ = 5.2, and *p*_down_ = 6.2 × 10^−7^, *z*_down_ = −4.9, upper and lower confidence interval (CI) crossing points within 1 s from air puff vs. baseline, Wilcoxon signed-rank test), go (D, *p*_up_ = 2.0 × 10^−7^, *z*_up_ = 5.2 and *p*_down_ = 3.4 × 10^−7^, *z*_down_ = −5.1) and no-go (E, *p*_up_ = 0.00015, *z*_up_ = 3.8 and *p*_down_ = 6.6 × 10^−8^, *z*_down_ = −5.4) trials respectively. **(F–H)** CCVs are computed after average response subtraction to reveal noise correlations. CCV in all trials (F, *p*_up_ = 0.0046, *z*_up_ = 2.8 and *p*_down_ = 5.9 × 10^−7^, *z*_down_ = −5.0), go (G, *p*_down_ = 3.5 × 10^−7^, *z*_down_ = −5.1) and no-go (H, *p*_up_ = 0.034, *z*_up_ = 2.1 and *p*_down_ = 5.6 × 10^−8^, *z*_down_ = −5.4) trials, respectively. Geometric mean firing rates (gmFR, mean ± standard error of the mean, SEM) are plotted in red on the right *y* axis. Lavender dashed lines represent 95% CI of the baseline CCV, and the gray dotted line represents the average trial-shuffled CCV (one shuffle per session). The shaded area represents SEM. Black and gray arrows mark the onset of the air puff and the auditory response cue, respectively. Wilcoxon signed-rank test was used for average upper and lower CI crossing points within 1 s from air puff vs. baseline for each session (*n* = 50 sessions).

Across 50 sessions, we observed a significant transient increase in total correlation between S1 and M2, followed by a prominent decrease in the canonical correlation value (CCV) relative to the baseline period (−0.25 to 0 s) (all trials: Fig 2C, *p*_up_ = 1.9 × 10^−7^, *z*_up_ = 5.2, and *p*_down_ = 6.2 × 10^−7^, *z*_down_ = −4.9; go trials: Fig 2D, *p*_up_ = 2.0 × 10^−7^, *z*_up_ = 5.2 and *p*_down_ = 3.4 × 10^−7^, *z*_down_ = −5.1; no-go trials: Fig 2E, *p*_up_ = 0.00015, *z*_up_ = 3.8 and *p*_down_ = 6.6 × 10^−8^, *z*_down_ = −5.4; upper and lower confidence interval (CI) crossing points up to 1 s post-stimulus versus baseline, Wilcoxon signed-rank test). CCV returned to baseline levels during the delay period and again dropped below the lower CI after the auditory response-cue presentation in all types of trials. Considering noise correlations, the transient CCV elevation was modest but significant when all trials were pooled and in no-go trials (all trials: Fig 2F, *p*_up_ = 0.0046, *z*_up_ = 2.8 and *p*_down_ = 5.9 × 10^−7^, *z*_down_ = −5.0; go trials: Fig 2G, *p*_down_ = 3.5 × 10^−7^, *z*_down_ = −5.1; no-go trials: Fig 2H, *p*_up_ = 0.034, *z*_up_ = 2.1 and *p*_down_ = 5.6 × 10^−8^, *z*_down_ = −5.4). Beyond this initial transient, temporal fluctuations in noise correlations closely mirrored those in total correlations. Because total correlation reflects both stimulus-driven shared tuning (signal) and trial-to-trial (noise) components, this similarity of temporal fluctuations indicates that the contribution of shared stimulus tuning between S1 and M2 rapidly diminished after the initial response. Thus, this implies that the two populations showed largely independent tuning once the transient sensory-evoked alignment subsided.

To further relate these interactions to behavior, we analyzed trial outcome types separately (S4 Fig). Correct trials (hit and CR) exhibited interaction patterns like those observed in go and no-go trials (S4A, S4B, S4E and S4F Fig), whereas error trials showed limited fluctuations in CCVs (S4C, S4D, S4G and S4H Fig). This contrast suggests that the observed interaction dynamics reflect goal-directed communication during successful performance, although limited sample size in the error trials precludes a fair comparison with the correct trials. The stronger and more sharply timed transient observed in total correlations relative to noise correlations suggests that the stimulus onset triggers a stimulus-gated S1–M2 coupling that exceeds spontaneous coupling and shared variability not directly attributable to stimulus drive. In other words, this early interaction reflects a rapid, stimulus-evoked alignment of population activity across the two regions, which is then followed by slower modulation of internally generated shared variability.

One possible explanation for the weak coupling during the delay period is variability in reaction times within and across sessions, which could blur together lick-evoked drops in CCV, and potential delay-period rises across trials. To account for this within-session variability, we realigned the spiking data to response onset (first lick) and recomputed CCV to examine delay-period coupling using total correlation (S4I and S4J Fig). We did not observe a significant increase in CCV before the first lick, although this could still reflect a mixture of stimulus-driven and delay-period coupling. To further isolate delay-period coupling from stimulus-evoked one, we excluded sessions with short reaction times (mRT ≤ 1.2 s), in which animals typically responded before the auditory cue. Even in sessions with long reaction times (mRT > 1.2 s, *n* = 33), CCV did not increase during the delay period, and it instead decreased below CI upon response onset (S4J Fig, total: *p*_down_ = 5.4 × 10^−7^, *z*_down_ = −5.0; noise: *p*_down_ = 7.8 × 10^−7^, *z*_down_ = −4.9). Notably, temporal fluctuations in CCV could not be explained by changes in firing rate. We assessed the geometric mean firing rate (gmFR) [57–59] across regions, which reflects the overall task-induced activity levels in the two populations irrespective of their trial-by-trial co-variation (Fig 2C–2H). Although gmFR across regions increased following response-cue onset, this was not accompanied by a corresponding rise in CCV, indicating that transient interareal coupling dynamics are not simply a reflection of global increases in population activity across regions.

Transient CCV increases may reflect behaviorally relevant communication between stimulus-selective populations within S1 and M2. Supporting this view, the magnitude of average CCV within 100 ms from stimulus onset was significantly correlated with behavioral discriminability (*d*-prime) when considering all trials (total: Fig 3A and 3D, *r* = 0.63, *p* = 2 × 10^−5^; noise: 3G and 3J, *r* = 0.53, *p* = 5 × 10^−4^, Pearson’s correlation) and when restricting analysis to go trials (total: Fig 3B and 3E, *r* = 0.60, p = 4.7 × 10^−5^, noise: 3H and 3K, *r* = 0.39, *p* = 0.015), with no-go trials showing no reliable relationship (total: Fig 3C and 3F, *r* = 0.14, *p* = 0.41; noise: 3I and 3L, *r* = −0.03, *p* = 0.86). This pattern indicates that the early, stimulus-locked interaction is closely tied to successful performance, particularly in trials that require an active decision to respond. By contrast, neither the post-stimulus decrease in CCV (0.1–0.3 s after stimulus onset) nor the later CCV increase during no-go trials (0.9–1.2 s) was significantly correlated with behavioral performance (total post-stimulus CCV: *p* = 0.1, *r* = 0.27; *p* = 0.052, *r* = 0.31 and *p* = 0.7, *r* = 0.056 for all trials, go and no-go trials; noise post-stimulus CCV: *p* = 0.1, *r* = 0.27; *p* = 0.053, *r* = 0.31 and *p* = 0.7, *r* = 0.055 for all trials, go and no-go trials; total later CCV: *p* = 0.06, *r* = 0.3; *p* = 0.3, *r* = 0.17, and *p* = 0.99, *r* = −0.002 for all trials, go and no-go respectively; noise later CCV: *p* = 0.06, *r* = 0.3; *p* = 0.3, *r* = 0.17, and *p* = 0.97, *r* = −0.005 for all trials, go and no-go respectively). These later fluctuations, therefore, likely index arousal-related, global changes in cortical state, rather than performance-sensitive communication. Especially, such decreases in correlated variability are consistent with the degradation of high-amplitude, synchronized activity often observed in quiet wakefulness [60–63].

**Fig 3:**
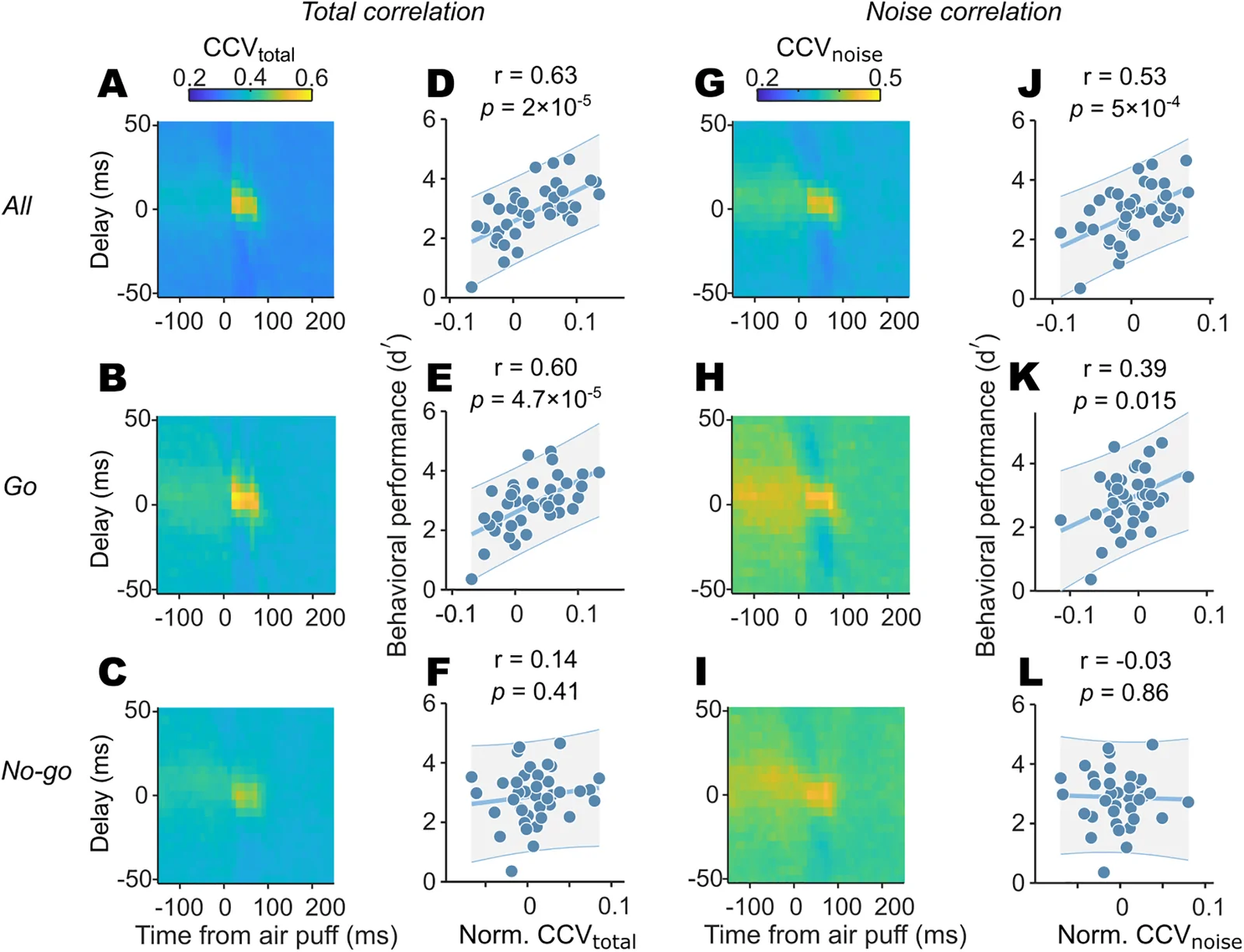
Transient interaction between S1 and M2 predicts behavioral performance. **(A–C)** Average total CCV maps across sessions showing a focal rise in total correlations shortly after air puff presentation across time bins and delays for all **(A)**, go **(B)**, and no-go trials, respectively. **(D–F)** Average of CCV_total_ within 100 ms from air puff and within 0 to 15 ms delays, is positively correlated with behavioral performance (*d*’) across 50 sessions for CCVs in all (D, *r* = 0.63, *p* = 2 × 10^−5^, Pearson’s correlation) and go (E, *r* = 0.60, *p* = 4.7 × 10^−5^) but not no-go (F, *r* = 0.14, *p* = 0.41) trials. **(G–I)** Average noise CCV maps across sessions showing a focal rise in noise correlations shortly after air puff presentation across time bins and delays for all **(G)**, go **(H)**, and no-go (I) trials, respectively. **(J–L)** Average of CCV_noise_ is positively correlated with behavioral performance (*d*’) across 50 sessions in all (J, *r* = 0.53, *p* = 5 × 10^−4^) and go (K, *r* = 0.39, *p* = 0.015) but not no-go (L, *r* = −0.03, *p* = 0.86) trials. Gray area marks 95% confidence intervals of the fitted lines. Each circle represents data from one session. Norm.: normalized to the baseline (250 ms before stimulus). The data underlying D–F and J–L can be found in https://doi.org/10.5281/zenodo.23338822 as 3DEFJKL.xlsx.

We further examined the relationship between transient interareal interactions and behavioral performance (d-prime) while accounting for session-to-session variation in neuronal selectivity using a linear mixed-effects model. In this model, behavioral performance was predicted by three factors: the average CCV within 100 ms of stimulus onset, the fraction of significantly selective neurons in M2, and the fraction of significantly selective neurons in S1, with subjects’ identity included as a random effect. Consistent with the Pearson’s correlation analyses described above, the model showed a significant positive coefficient for average CCV (*p* = 0.001, *t* = 3.6 and *df* = 31 for total correlation using all trials; *p* = 0.004, *t* = 3.1 and *df* = 31 for noise correlation using all trials, n = 50 sessions from 18 mice). In contrast, population selectivity in either area did not significantly predict behavioral performance (total: M2: *p* = 0.35, *t* = −0.94, S1: *p* = 0.34, *t* = −0.96; noise: M2: *p* = 0.97, *t* = −0.04, S1: *p* = 0.69, *t* = 0.41, *df* = 31). No significant interactions were found among the three predictors for either total or noise correlation. Even in reduced single-predictor models, population selectivity in S1 or M2 alone failed to predict behavioral performance (S1: *p* = 0.27, *t* = 1.1, M2: *p* = 0.34, *t* = 0.96, *df* = 37). These results indicate that the transient interareal interactions between S1 and M2 are a robust predictor of behavioral performance, whereas variability in population selectivity likely arises from receptive field variations among sessions and does not account for behavioral variability.

We consolidated these results with a trial-resolved analysis to test whether moment-to-moment fluctuations in inter-areal coupling predict the behavioral outcome of each trial. To minimize the influence of fluctuations in arousal or task engagement, we first estimated the animal’s moment-to-moment performance level throughout the session using a local hit-rate measure and excluded trials occurring during periods of very low engagement (local hit rate < 0.2). This procedure preferentially removed trials in which animals were likely disengaged from the task, thereby reducing potential confounds related to arousal and task engagement. Using the remaining engaged trials, we computed trial-level coupling; For each trial, we quantified coupling strength as the average correlation between the first pair of canonical variables within 100 ms of the air puff in either the feedforward (FF: 2–20 ms) or feedback (FB: −20 to −2 ms) direction. We then applied a binomial mixed-effects model with trial outcome (correct versus incorrect) as the response variable, trial-level coupling as a fixed effect, and sessions and subjects as random effects.

In high-selectivity sessions, trial-level coupling significantly predicted the trial outcome only in the feedforward direction (FF: *p* = 0.0045, *t = 2.84*, FB: *p* = 0.24, *t* = 1.2, *df* = 5,242, 25 sessions from 12 mice). In low-selectivity sessions, there was no significant association between trial-level coupling and trial outcome in either direction (FF: *p* = 0.80, *t* = −0.26, FB: *p* = 0.53, *t* = 0.62, *df* = 5,065, 25 sessions from 13 mice). Consistent with this result, including selectivity (*S1sel*) as a fixed-effect predictor revealed a significant, positive interaction between trial-level feedforward coupling and the fraction of selective S1 neurons in each session (FF: *p*_*coupling ×*_ *_S1_*_*sel*_ = 0.017, *t*_*coupling ×*_ *_S1_*_*sel*_ = 2.38, *p*_*coupling*_ = 0.44, *t*_*coupling*_ = −0.76, *p*_*S1sel*_ = 0.35, *t*_*S1sel*_ = −0.93, FB: *p*_*coupling×S1sel*_ = 0.21, *t*_*coupling × S1sel*_ = 1.26, *p*_*coupling*_ = 0.92, *t*_*coupling*_ = −0.11, *p*_*S1sel*_ = 0.89, *t*_*S1sel*_ = 0.14, *df* = 10,307, 50 sessions, S5 Fig), while selectivity in M2 (*M2sel*) did not interact with the trial-level coupling (FF: *p*_*coupling × M2sel*_ = 0.90, *t*_*coupling × M2sel*_ = 0.13, FB: *p*_*coupling × M2sel*_ = 0.46, *t*_*coupling × M2sel*_ = −0.74). Together, these analyses indicate that stronger early feedforward S1–M2 coupling is associated with successful behavior within trials, specifically when sensory representations in S1 are strong.

Because the canonical weights used for this analysis were estimated from all trials within each session, we repeated it with 10-fold cross-validation, estimating weights from 90% of trials and applying them to the held-out 10%. For total coupling, the association between feedforward coupling and trial outcome was preserved (go trials of high-selectivity sessions: *p* = 0.006, *t* = 2.74, *df* = 2,846). For noise coupling, cross-validation reduced the estimated coupling roughly 5-fold (mean 0.184 versus 0.038) and the association with trial outcome was no longer detectable (*p* = 0.92, *t* = −0.10, *df* = 2,847), indicating that the canonical subspace estimated from residual activity within a single session does not generalize to held-out trials. The trial-level noise-coupling results reported here, including the delay-period analyses below, should therefore be read as a within-session characterization rather than an independently validated prediction.

To test the possibility of a link between trial-level coupling and behavioral state, we regressed the trial-level coupling with a proxy of state. Although pupil dynamics around whisker stimulus are likely too slow to be used as a predictor of the transient coupling [64], reflexive movements of the facial area in response to stimulation [65] could be used as a proxy of the state with sufficiently short latency. We therefore included an analysis incorporating the motion index [66] as a proxy of state to predict trial-level coupling in high-selectivity sessions where transient trial-level coupling predicted the outcome. Motion index quickly increased upon air puff presentation, indicating reflexive movements in response to the puff (S6A Fig) as reported elsewhere [65]. We asked whether trial-level coupling in three distinct periods can be related to the motion index as a state variable during 3 periods of: (1) baseline; −100 to 0 ms from air puff, (2) transient; 0 to 100 ms from air puff and (3) decorrelation; 120 to 220 ms from air puff. Trial-level coupling was computed as detailed previously. S6B Fig shows the relationship between motion index and trial-level coupling averaged over the first 100 ms after air puff onset (5,244 trials from 25 high-selectivity sessions). After z-scoring both variables within each session, no significant correlation was observed (*r* = −0.02, *p* = 0.145, Pearson’s correlation).

Using a linear mixed-effects model with session and subject included as random effects, we tested whether trial-level coupling strength is predictable from the motion index during each of the three periods. Across all trials from high-selectivity sessions, neither feedforward nor feedback coupling showed a significant association with motion index during any of the three periods (baseline: FF: *p* = 0.53, *t* = −0.62, FB: *p* = 0.87, *t* = 0.16; transient: FF: *p* = 0.19, *t* = 1.3, FB: *p* = 0.91, *t* = 0.11; decorrelation: FF: *p* = 0.87, *t* = −0.17, FB: *p* = 0.23, *t* = 1.2, *df* = 5,242). Thus, trial-to-trial fluctuations in movement state did not predict fluctuations in inter-areal coupling strength despite the rise of both variables during the first 100 ms of the air puff based on the grand average data (S6C Fig). We further examined whether the motion index during the transient period contributed to behavioral performance, either independently or through an interaction with trial-level coupling strength. Trial-level motion index alone was not associated with performance (*p* = 0.87, *t* = −0.16, linear mixed-effects, *df* = 5,242). When both motion index and coupling strength were included in the model, only feedforward coupling during the transient epoch remained a significant predictor of behavioral performance. Neither motion index nor its interaction with coupling contributed significantly, and no significant effects were observed for feedback coupling (FF: *p*_*motion*_ = 0.68, *t*_*motion*_ = −0.42, *p*_*motion x coupling*_ = 0.71, *t*_*motion × coupling*_ = 0.37, *p*_*coupling*_ = 0.016, *t*_*coupling*_ = 2.4, FB: *p*_*motion*_ = 0.24, *t*_*motion*_ = −1.2, *p*_*motion x coupling*_ = 0.25, *t*_*motion × coupling*_ = 1.2, *p*_*coupling*_ = 0.61, *t*_*coupling*_ = 0.52, *df* = 5,240). These results indicate that trial-to-trial fluctuations in movement-related state do not account for the observed variability in inter-areal coupling. Instead, they support the interpretation that the transient increase in S1–M2 coupling reflects specific functional interactions between the two regions rather than nonspecific modulation due to motion-related activity.

We found no significant rise in the delay-period coupling even when the data was aligned to the response time (S4J Fig). However, it is possible that S1–M2 coupling in the delay period is highly variable from trial to trial in a way that could predict performance or response time, while it is masked by the session-level analysis. To address this possibility, we investigated whether trial-level delay-period coupling could predict future licks (i.e., reaction time) or trial outcome (correctness). We computed trial-level coupling as previously described, except that we chose larger, non-overlapping windows to account for potentially slower coupling (window size = 250 ms, step = 250 ms, bin size = 2 ms), using trials with the reaction time between 1.25 and 6.25 s. First, we tested whether the trial-level delay period coupling (noise correlations, 0.25–1.25 s from air puff) can predict the trial outcome in a binomial mixed-effects model with session and subjects as random effects. We did not find a significant association between trial-level, delay-period coupling and trial outcome in either direction (FF: *p* = 0.13, *t =* 1.5, FB: *p* = 0.49, *t* = 0.70, *df* = 6,853, 36 sessions from 16 mice). Including S1 selectivity (*S1sel*) as a fixed effect did not change the story (FF: *p*_*coupling*_ = 0.54, *t*_*coupling*_ = 0.61, *p*_*S1sel*_ = 0.73, *t*_*S1sel*_ = −0.35, *p*_*coupling ×*_ *_S1_*_*Sel*_ = 0.78, *t*_*coupling × S1sel*_ = 0.28, FB: *p*_*coupling*_ = 0.81, *t*_*coupling*_ = −0.24, *p*_*S1Sel*_ = 0.46, *t*_*S1sel*_ = −0.73, *p*_*coupling × S1Sel*_ = 0.40, *t*_*coupling × S1sel*_ = 0.83, *df* = 6,851).

Furthermore, in a linear mixed-effects model where trial-level, delay-period coupling and selectivity were used to predict reaction time, we found no significant association (FF: *p*_*coupling*_ = 0.63, *t*_*coupling*_ = 0.49, *p*_*S1Sel*_ = 0.33, *t*_*S1sel*_ = −0.98, *p*_coupling × S1Sel_ = 0.93, *t*_coupling × S1Sel_ = 0.09, FB: *p*_*coupling*_ = 0.059, *t*_*coupling*_ = 1.9, *p*_*S1Sel*_ = 0.64, *t*_*S1Sel*_ = −0.46, *p*_*coupling × S1Sel*_ = 0.59, *t*_*coupling × S1Sel*_ = −0.53, *df* = 4,274, 36 sessions from 16 mice). However, trial-level delay-period coupling as the sole fixed-effect variable significantly predicted reaction time in the feedback direction (FB: *p* = 0.020, *t* = 2.3, FF: *p* = 0.36, *t* = 0.92, *df* = 4,276) with a positive weight (*β* = 0.68). This relationship was maintained when using broader feedback delay (−50 to −20 ms) instead of the original feedback delays (−20 to −2 ms) for trial-level coupling, indicating that it could arise from indirect feedback from M2 to S1. Indeed, this relationship was not limited to this period and these sessions with longer reaction times. Across all sessions, a significant positive association existed between trial-level coupling and reaction time (−100–0 ms from air puff: *p* = 0.043, *t* = 2.0; 0–200 ms from air puff: *p* = 0.003, *t* = 2.9, *df* = 6,213, 50 sessions). Its positive relationship with reaction time indicates that the feedback from M2 to S1 may help to maintain an interacting loop between sensory and motor regions, to delay decision until further evidence (e.g., response cue) is available, or serve as a top-down learning signal [67] which relays feedback based on the recent decisions and sensory cues.

Overall, beyond the cross-session correlation analysis, these trial-resolved analyses indicate that stronger early feedforward S1–M2 coupling predicts successful behavior within trials, specifically when sensory information is robustly represented in S1, although the noise-coupling version of this analysis relies on subspaces estimated within session and was not recovered under cross-validation. Furthermore, our analyses showed that trial-to-trial fluctuations were not associated with fluctuations in motion-related behavioral state.

### S1 and M2 exchange stimulus selectivity information in brief interactions

Interareal interactions reflecting direct communications must occur within short delays between the activities of the two regions [43]. To examine the directionality of these transient interactions during the air puff, we calculated feedforward and feedback components in short delays using all trials in each session for CCA (<15 ms, Fig 4A–4C). On average, the transient rise of CCV was significant only in the feedforward direction (noise: S1 → M2, Fig 4B, *p*_up_ = 0.0003, *z*_up_ = 3.7, Wilcoxon signed-rank test on upper CI crossing points relative to the baseline window, −50 ms to 0). In the feedback direction, CCV instead dropped significantly below baseline during the air puff, followed by a transient rebound to baseline and a subsequent drop (noise: M2 → S1, Fig 4C, *p*_down_ = 5.6 × 10^−5^, *z*_down_ = −4.0, Wilcoxon signed-rank test on lower CI crossing points (average) relative to baseline, n = 50 sessions).

**Fig 4:**
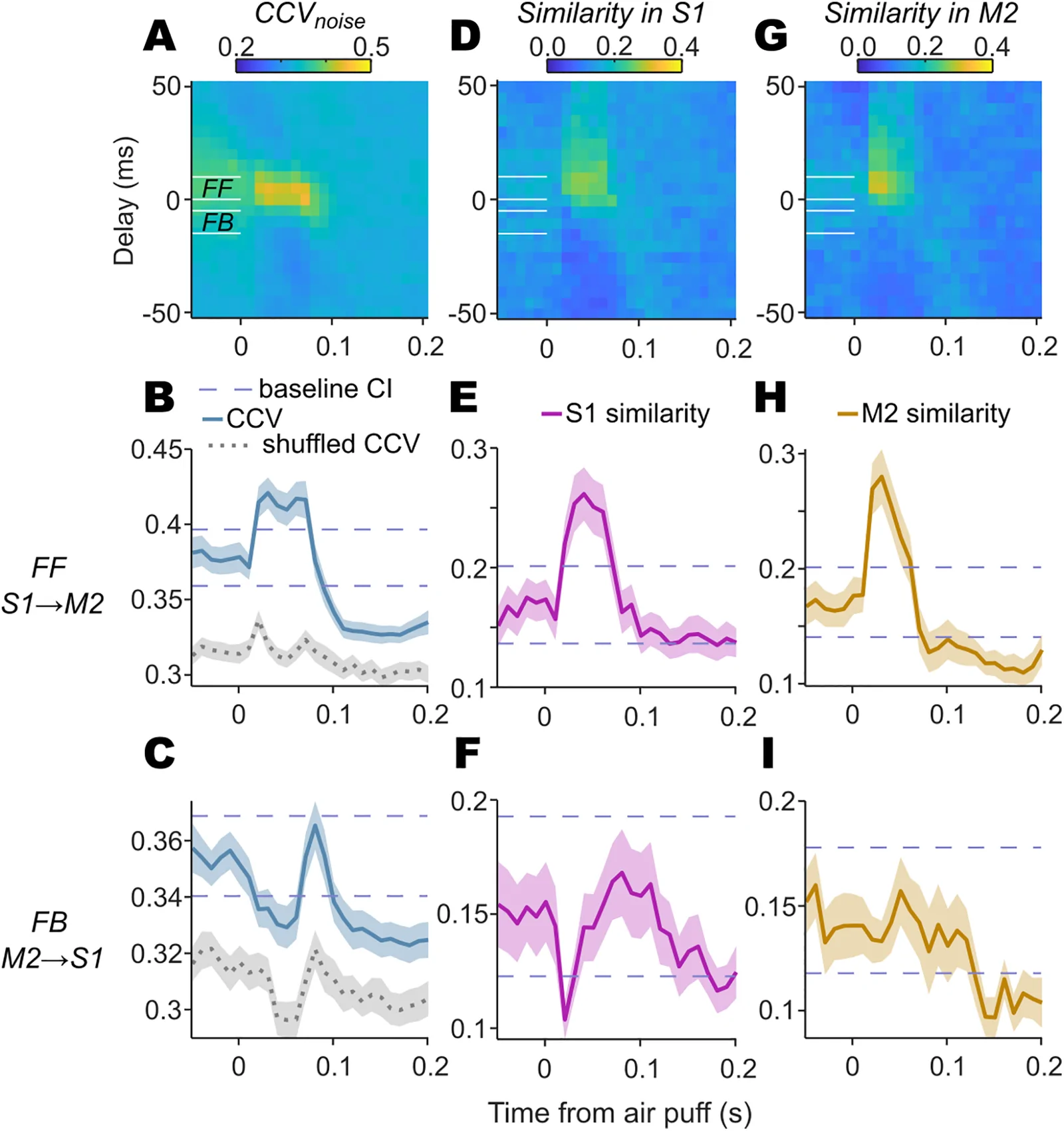
Communication subspaces of the two areas align with the local stimulus coding subspace in noise correlations. **(A–C)** Average CCV_noise_ across sessions in each time window and delay, using all trials in each session (A), showing significant feedforward (B, S1 → M2: *p*_up_ = 0.0003, *z*_up_ = 3.7 and *p*_down_ = 1.5 × 10^−6^, *z*_down_ = −4.8, upper and lower CI crossing points against baseline, Wilcoxon signed-rank test) but not feedback (C, M2 → S1: *p*_down_ = 5.6 × 10^−5^, *z*_down_ = −4.0) component of the transiently enhanced interaction. **(D–F)** Average magnitude of similarity between ρd and cd_S1_ (henceforth “similarity”) in each time window across sessions using all trials in each session, showing a similar feedforward component (E, S1 → M2: *p*_up_ = 1.5 × 10^−5^, *z*_up_ = 4.3, *p*_down_ = 0.1, *z*_down_ = −1.6) with no significantly enhanced feedback component (F, M2 → S1: *p*_down_ = 0.019, *z*_down_ = −2.3). **(G–I)** Average magnitude of similarity between ρd and cd_M2_ computed using all trials in each session (H, S1 → M2: *p*_up_ = 6.8 × 10^−5^, *z*_up_ = 4.0 and *p*_down_ = 0.002, *z*_down_ = −3.1) with no significantly enhanced feedback component (I, M2 → S1: *p*_down_ = 0.014, *z*_down_ = −2.5). Average data are represented as mean ± SEM. White lines indicate delay intervals selected for feedforward (FF) and feedback (FB) components. Lavender dashed lines mark 95% confidence interval of the baseline. Gray dotted line represents shuffled CCV. Wilcoxon signed-rank test was used to test the differences between average upper or lower CI crossing points within 200 ms from the air puff onset versus baseline (*n* = 50 sessions).

To test whether the observed interactions carried stimulus-related information, we computed a coding direction (cd) [68,69] using all trials in each session, during the first 100 ms of the stimulus presentation by subtracting the mean right-trial activity vector across neurons from the mean left-trial vector within each area. The resulting vector captures the population pattern, distinguishing between the two types of stimuli. Next, we computed another vector, a communication subspace (ρd), as the weight of the first canonical dimension in each area. Unlike cd, which is fixed once defined, ρd is time-varying and represents the axis along which S1 and M2 activities are most correlated at each moment. We quantified the similarity between cd and ρd by calculating the cosine of the angle between the two vectors (i.e., projection magnitude of unit vectors, henceforth called “similarity”, Fig 4D and 4G). In the feedforward direction, both S1 and M2 similarity magnitudes rose significantly above baseline in parallel with the CCV (Fig 4E and 4H: *p*_up_ = 1.5 × 10^−5^, *z*_*up*_ = 4.3 and *p*_*up*_ = 6.8 × 10^−5^, *z*_*up*_ = 4.0, respectively). However, in the feedback direction, there was no significant rise in the similarity in either area (Fig 4F and 4I). Total correlations instead revealed significant coupling in both directions (S7A–S7I Fig). However, interactions were consistently weaker in the feedback direction (total: CCV: *p* = 5.7 × 10^−9^, *z* = 5.8, S1 similarity: *p* = 3.4 × 10^−5^, *z =* 4.1, M2 similarity: *p* = 1.0 × 10^−5^, *z* = 4.4; noise: CCV: *p* = 1.3 × 10^−8^, *z* = 5.7, S1 similarity: *p* = 2.2 × 10^−5^, *z =* 4.2, M2 similarity: *p* = 3.1 × 10^−6^, *z* = 4.7, Wilcoxon signed-rank test within 100 ms of air puff).

Is the early communication pattern originating from S1 required for successful task performance, and do these patterns causally shape early stimulus selectivity in M2? To address these questions, we transiently suppressed S1 during the early sensory period using optogenetics in PV-ChR2 mice (S8A Fig). Suppression of the right S1 significantly reduced behavioral discriminability (d-prime) compared with both the control condition and left S1 suppression (S8B Fig: *p* = 0.015, *f* = 9.2, *df* = 2; Control versus Right S1: *p* = 0.019, Left S1 versus Right S1: *p* = 0.042, 4 mice, repeated-measure ANOVA followed by Fisher’s LSD tests). Furthermore, recordings from the right M2 identified 147 neurons exhibiting significant stimulus selectivity within the first 100 ms after the air puff. Brief S1 suppression significantly decreased this early M2 selectivity (S8C Fig: *p* = 0.0017, *t* = −3.17, *df* = 292, 9 sessions from 4 mice, linear mixed effects). These results demonstrate that early S1 activity is required for both the emergence of robust stimulus selectivity in M2 and successful performance of the go/no-go task.

To assess whether the inclusion of multi-unit activity influenced the coupling dynamics, we repeated this analysis using the high-quality fraction of the units in both areas. Because a high inter-spike-interval (ISI) violation rate is commonly interpreted as evidence of poor unit isolation or contamination from spikes belonging to multiple neurons, we repeated the CCA analysis after excluding units with a high ISI violation rate and retaining ~80% of the units in each area (S9A Fig). This restriction enriches the dataset for better-isolated single units and reduces the likelihood that the observed delay reflects multiunit contamination or spike-sorting artifacts. Using the high-quality fraction of the units in both areas (~80%), we found highly consistent results regarding the dynamics of coupling across two areas for both types of correlation measures (CCV_noise_: S9B Fig and CCV_total_: S9C Fig).

Our analysis of the coding-communication alignment utilized the same trials for ***ρd*** and ***cd*** estimations and therefore is prone to overfitting (i.e., alignment may arise from large co-fluctuations in limited trials). We therefore additionally performed a split-half cross-validation to independently estimate the communication and coding axes. We reproduced our similarity results in both feedforward and feedback directions using independent trials for ***ρd*** and ***cd***. Consistent with the original results, similarity in both regions increased significantly above baseline in the feedforward direction using either noise or total correlations (noise: S10A and S10B Fig, S1 similarity: *p*_*up*_ = 0.003, *z*_*up*_ = 2.9, M2 similarity: *p*_*up*_ = 0.033, *z*_*up*_ = 2.1; total, S10D and S10E Fig, S1 similarity: *p*_*up*_ = 5.8 × 10^−6^, *z*_*up*_ = 4.5, M2 similarity: *p*_*up*_ = 5.8 × 10^−6^, *z*_*up*_ = 4.5, upper confidence interval (CI) crossing points up to 100 ms post-stimulus versus baseline, Wilcoxon signed-rank test). In the feedback direction (S10C and S10F Fig), only total correlations showed a subtle but significant increase in similarity in both areas (S10F Fig, S1 similarity: *p*_*up*_ = 0.03, *z*_*up*_ = 2.2, M2 similarity: *p*_*up*_ = 0.01, *z*_*up*_ = 2.5). This cross-validated analysis shows that the alignment of the shared and local coding subspaces is generalizable to independent trials and therefore unlikely to arise from large fluctuations in limited trials. In other words, the shared activity captures coding properties of the population regardless of trial-to-trial variability in response. Furthermore, we repeated the CCA analysis using 10-fold cross-validation to control for potential overfitting of CCA itself. In each fold, trials were randomly divided into training (90%) and test (10%) sets. CCA weights were estimated using only the training trials, and the resulting canonical coefficients were then applied to the held-out test trials to compute the CCVs. Final CCVs were obtained by averaging across folds (S11 Fig: average cross-validated CCVs across 50 sessions). This cross-validated analysis produced results highly consistent with the original non-cross-validated CCA, including communication timing and coupling magnitude across delays. Overall, these analyses demonstrate that the observed feedforward communication and its alignment with the coding subspace are robust and are unlikely to arise from overfitting.

Our similarity measure is based on absolute values. We observed considerable sign flips from one time window to another within sessions and across sessions (Example sessions in S12A Fig). Importantly, such sign changes do not necessarily indicate a loss of alignment between coding and communication subspaces. Because cosine similarity is sensitive to vector orientation, a reversal in the dominant activity pattern between interacting populations can invert the sign while still preserving a strong subspace relationship. Thus, both positive and negative cosine values can reflect structured alignment between stimulus-coding and communication axes. To directly test whether sign ambiguity influenced our conclusions, we repeated the analysis using element-wise products of the signed similarity matrices from S1 and M2. The resulting map was highly similar to those obtained using absolute cosine similarity across sessions (S12B Fig, FF: *p*_*up*_ = 5.9 × 10^−7^, *z*_*up*_ = 5.0, FB: *p*_down_ = 0.18, *z*_down_ = −1.3, *n* = 50 sessions, upper and lower confidence interval (CI) crossing points up to 100 ms post-stimulus versus baseline, Wilcoxon signed-rank test), indicating that the reported effects were not driven by arbitrary sign flips. Rather, the transient increases in similarity reflect robust alignment between the stimulus-coding subspace and the communication subspace during the brief interaction window. Notably, the feedforward component of the product of similarity was significantly correlated with the sessions’ S1 selectivity, further emphasizing the specificity of the communication-coding alignment (0–100 ms, FF: *p* = 6.7 × 10^−5^, *t* = 4.4, FB: *p* = 0.63, *t* = 0.48, *df* = 48, linear mixed-effects).

Feedback interactions were observed only in total correlations. Indeed, in noise correlations, the feedback effect emerged as a small, delayed peak (50–100 ms) superimposed on the stimulus-evoked desynchronization. Notably, this enhancement was not associated with a significant rise in ρd**-**cd similarities. During feedforward interactions, ρd and cd were consistently aligned, implying alignment of signal and noise axes [41,70]. This is evident in consistently enhanced similarity values in both total and noise correlations following the air puff. In contrast, in the feedback direction of noise interactions, the lack of alignment between ρd and cd implies that signal and noise axes are independent [41,70–72]. Therefore, the enhanced feedback in total correlations and the corresponding similarities indicate dominant co-fluctuation along a signal axis that is independent of noise correlations. Nevertheless, the presence of this feedback component is consistent with previous reports of feedback signaling between somatosensory and frontal areas [22,73].

These findings reveal that S1 and M2 quickly share task-relevant sensory information within a brief, stimulus-locked window, with communication confined to the earliest stimulus-evoked population pattern, rather than sustaining prolonged or recurrent communication. Moreover, the directionality of signal and noise correlations collectively suggests that a trial-to-trial interaction occurs predominantly in the feedforward direction.

To confirm that the observed similarities reflected stimulus-related rather than response-related activity, we tested the temporal consistency of cd vectors [68] computed at different time points (S13A and S13B Fig). In both areas, the cd defined during the transient interaction time window (0–100 ms after air-puff onset) showed low consistency with cds derived from later time windows, indicating that early patterns uniquely encoded stimulus identity rather than the animals’ behavioral response that emerges closer to the choice period. We next computed similarity values based on a late cd (1–1.1 s), corresponding to the late delay period. These similarity values were significantly lower than those based on the early cd in both areas, and this effect was specific to the feedforward direction (S13C–S13F Fig, total FF: S1_cd_ versus S1_late cd_: *p* = 9.4 × 10^−5^, *z* = 3.9, M2_cd_ versus M2_late cd_: *p* = 1.0 × 10^−7^, *z* = 5.3; total FB: S1_cd_ versus S1_late cd_: *p* = 0.16, *z* = 1.4, M2_cd_ versus M2_late cd_: *p* = 0.15, *z* = 1.4; noise FF: S1_cd_ versus S1_late cd_: *p* = 0.0004, *z* = 3.6, M2_cd_ versus M2_late cd_: *p* = 1.1 × 10^−7^, *z* = 5.3; noise FB: S1_cd_ versus S1_late cd_: *p* = 0.88, *z* = 0.1, M2_cd_ versus M2_late cd_: *p* = 0.68, *z* = 0.4, Wilcoxon signed-rank tests, *n* = 50 sessions).

These results confirm that the early feedforward interactions between S1 and M2 contain transient sensory coding patterns relevant for task performance but do not persist into later, preparatory, or action-related periods.

### A short delay of S1–M2 interactions suggests a potentially direct, feedforward coupling

To further probe the directionality of S1 and M2 interactions, we examined the temporal delay of interacting signals. Averaging CCV across sessions as a function of delay revealed a feedforward bias, with stronger coupling at positive delays (Fig 5A–5C, top). In many sessions, CCV peaked at a short and positive delay, indicating rapid transmission of sensory information from S1 to M2, compatible with a potentially direct feedforward pathway (Fig 5C, top). Correspondingly, the magnitude of cosine similarity in both regions also exhibited a feedforward bias in most of the sessions (Fig 5A–5C, middle and bottom). The feedforward bias of the CCV peak was especially prominent in high-selectivity sessions (Fig 5D, top, median peak delay: 0 ms versus +5 ms for low- and high-selectivity sessions, respectively, noise: *p* = 0.018, *t* = 2.4, total: *p* = 0.046, *t* = 2.0, *df* = 48, linear mixed-effects using each sessions selectivity as a fixed-effect and subjects’ identity as a random effect).

**Fig 5:**
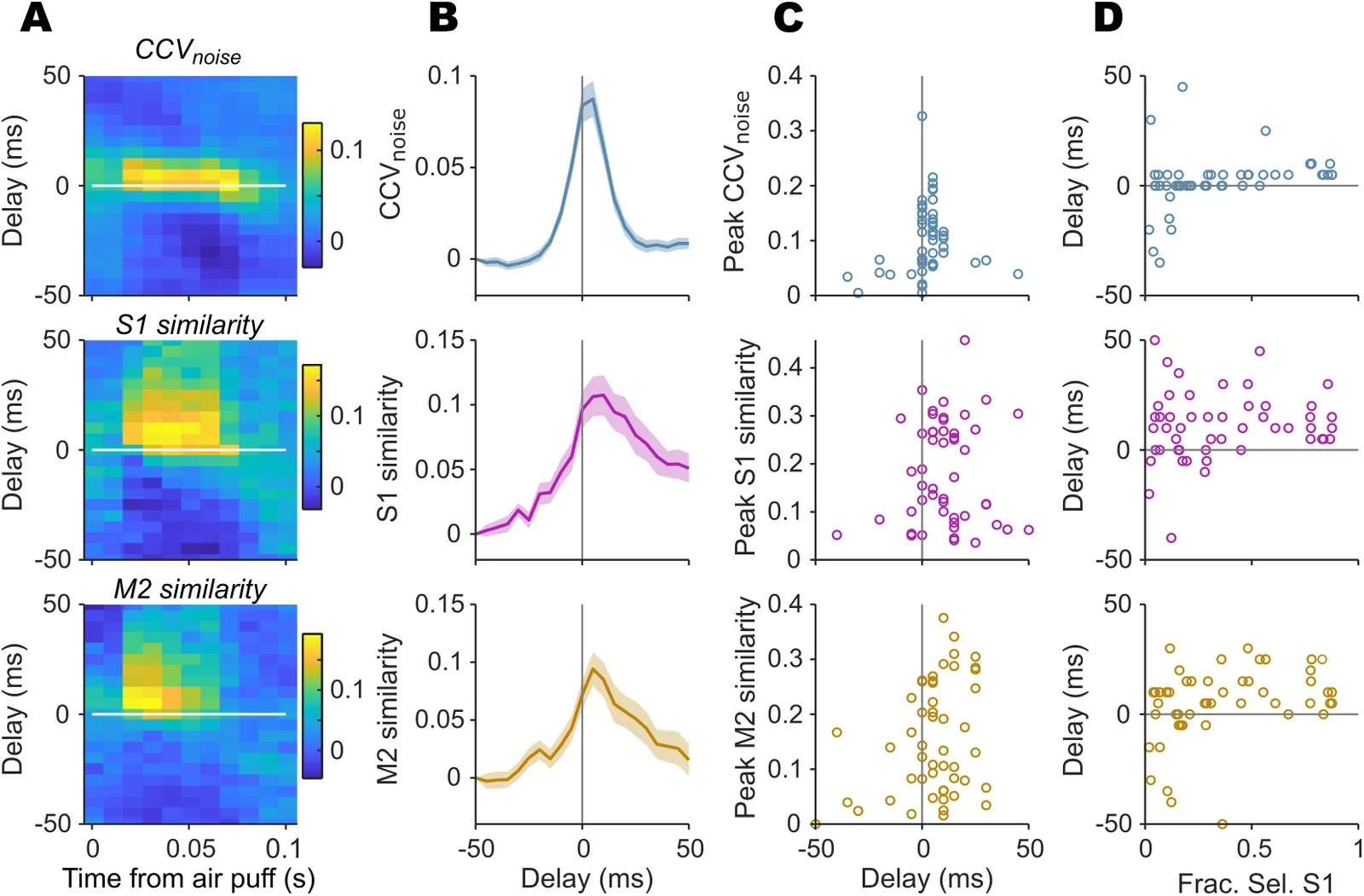
Peak of interactions confirms a feedforward bias across sessions. **(A**) Baseline-corrected CCV_noise_ profile (top), S1 similarity (middle), and M2 similarity (bottom) averaged across sessions. **(B)** CCV_noise_ (top), S1 similarity (middle), and M2 similarity (bottom) averaged (± SEM) across sessions within 100 ms from air puff, for each delay. **(C)** Delay of the peak in CCV_noise_ (top), S1 similarity (middle), and M2 similarity (bottom) for each session within 100 ms from the air puff. **(D)** Delay of the peaks as a function of population selectivity for CCV_noise_ (top), S1 similarity (middle), and M2 similarity (bottom). Each circle in C and D represents a session (*n* = 50).

Considering total correlations, similarities showed a more dynamic temporal profile (S14A Fig); both S1 and M2 similarities showed significantly different peak time between early (0–50 ms) and late (50–100 ms) post-stimulus time windows (S14B and S14C Fig, S1: *p* = 0.018, *z* = 2.4, M2: *p* = 0.002, *z* = 3.1 CCV: *p* = 0.875, *z* = 0.16, Wilcoxon signed-rank test). The peak of M2 similarity prominently shifted from a feedforward delay in the early window (median = +5 ms) to a feedback delay in the late window (−15 ms). This temporal shift was invariant to the choice of cd window around stimulus onset and reproduced when a cd was defined from 100 to 200 ms after stimulus onset (early window: +7.5 ms; late window: −20 ms, *p* = 0.001, *z* = 3.3, Wilcoxon signed-rank test), indicating that the similarity profile emerges due to stimulus selectivity and not merely due to the choice of cd window. Overall, these results reveal rapid, stimulus-selective feedforward communication from S1 to M2 within 100 ms of the air puff onset. A weaker, delayed feedback communication reflected M2 stimulus selectivity patterns but likely arose through indirect pathways. Notably, a consistent feedforward bias was also observed during inter-trial intervals (S14D–S14G Fig) and in CCVs of second to fourth canonical dimensions, even though these dimensions did not resemble stimulus coding patterns (Figs 6A–6C and S15A–S15C). This suggests that, in parallel to stimulus identity, additional sensorimotor information is likely transferred from S1 to M2 and may contribute to shaping behavioral output.

**Fig 6:**
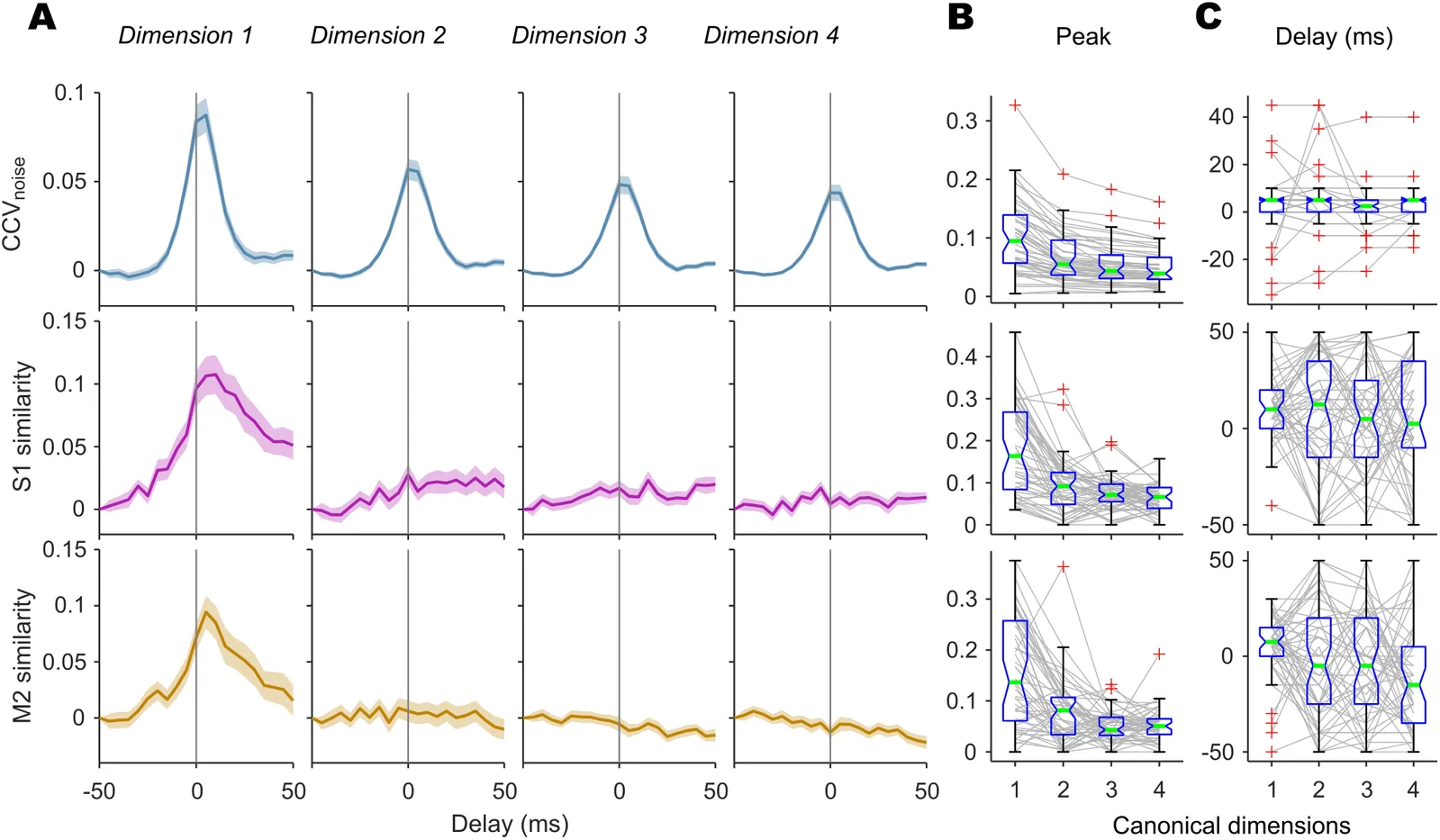
Consistent feedforward interactions across subsequent canonical dimensions. **(A**) Average (±SEM) of CCV_noise_ (top row), S1 similarity (middle row), and M2 similarity (bottom row) across canonical dimensions (columns). **(B)** Peak magnitude of CCV_noise_ (top), S1 similarity (middle) and M2 similarity (bottom) across 4 canonical dimensions. **(C)** Delay of the peak in CCV_noise_ (top), S1 (middle), and M2 (bottom) similarity across canonical dimensions. Each gray line in B and C represents one session. Boxplots represent the median (green) and interquartile range (IQR, blue) of the data in each dimension and the whiskers extend to the last data point not considered an outlier. Red plus signs represent outliers (1.5 times IQR below and above the first and the third quartiles, respectively). Notches represent 95% confidence intervals of medians. The data underlying B and C can be found in https://doi.org/10.5281/zenodo.23338822 as 6BC.xlsx.

We additionally tested whether the delay-period interactions potentially align with a choice subspace of long RT sessions. For this analysis, we included long RT trials in which licking occurred within an extended response window (1.2–5 s from air puff) and excluded trials with premature responses (0–1.2 s from air puff). A choice coding subspace was then computed in each area using neural activity during the delay period (0.25–1 s after air puff onset). We derived one choice coding vector for each consecutive 250 ms window (4 total analysis windows), analogous to the stimulus coding direction ***cd***. We then repeated the CCA analyses and computed the similarity between the communication subspace and the choice coding subspace at each 250-ms time window and averaged them across all 4 windows and all sessions. Across all the first 4 canonical dimensions, similarities were flat, near-zero for all delays and for both total and noise correlations (S16 Fig), indicating that the delay-period communication subspace did not align with the choice-coding subspace.

To further consolidate these results, we repeated the CCA with 1-ms binning to more precisely localize the peak of CCVs (S17A–S17C Fig). Across sessions, peak CCV delay systematically decreased as the fraction of selective S1 neurons increased (S17D Fig, median = +3 ms). CCV peak magnitude dropped across canonical dimensions (*dim*) regardless of S1 selectivity (*S1sel*) (S17E, S17F, S17H and S17I Fig, top; *p*_dim_ = 7.1 × 10^−15^, *t*_dim_ = −8.1, *p*_S1sel_ = 0.29, *t*_S1sel_ = 1.07, *df* = 392, linear mixed-effects, 50 sessions from 18 mice), the absolute peak delay depended on both dimension and selectivity (S17G and S17J Fig, top; *p*_dim_ = 0.0013, *t*_dim_ = −3.2, *p*_S1sel_ = 0.0003, *t*_S1sel_ = −3.6, *p*_*dim × S1sel*_ = 0.043, *t*_dim × S1sel_ = 2.03, *df* = 392, linear mixed-effects). High-selectivity sessions had a median absolute peak delay of 4 ms in the first dimension, indicating a rapid feedforward [74,75] coupling from S1 to M2 (S17G Fig, top), whereas low-selectivity sessions showed longer delays overall (S17J Fig, top). To ensure that these delays did not arise from slow or indirect shared dynamics [76], we computed a jittered-CCV [43] and subtracted it to obtain a jitter-corrected CCV. This revealed that the first canonical dimension retained the short median absolute peak delay (4 ms) after correction (S17G Fig, bottom), with no significant interaction between correction and selectivity (*p*_correction_ = 0.50, *t*_correction_ = −0.68, *p*_correction × S1sel_ = 0.71, *t*_*correction × S1sel*_ = 0.37, *df* = 392, linear mixed effects).

These results provide convergent evidence that sensory information is transmitted from selective S1 populations to M2 via a fast, potentially direct feedforward pathway, and that this short-delay coupling depends on the degree of stimulus selectivity in S1.

We performed control and alternative analyses to test the reliability of peak delay estimations across a range of parameters and conditions. First, the average CCV maps in Fig 3 suggest that the positive delays are not uniformly produced across all conditions by the CCA analysis itself; in no-go trials (Fig 3C and 3I), the interaction peak appears at zero delay, despite being analyzed with the identical CCA procedures. We, therefore, considered the peak delays of go and no-go trials separately (S18A–S18H Fig). This analysis clearly shows that no-go trials exhibited delay distributions centered at zero (median = 0 ms, *p* = 0.17*, z* = 1.37, Wilcoxon signed rank test, *n* = 50 sessions), whereas go trials showed a significantly positive delay (median = +5 ms, *p* = 0.0006, *z* = 3.43, *n* = 50 sessions). Thus, the analysis framework is capable of distinguishing near-synchronous interactions from delayed interactions, arguing against the possibility that the observed positive delay in go trials arises trivially from temporal autocorrelation, binning, or shared-input structure alone.

Furthermore, we simulated a simple inter-areal interaction to validate the CCA peak delay estimator. We modeled the spiking activity of two neuronal populations with a simple Poisson process (70 neurons each, 150 trials), where a delayed coupling between the two populations was increased 50-fold from a negligible baseline during a predefined 50-ms interaction period (green shading, delay = 10 ms, S18I and S18J Fig). We then applied the identical time-shifted CCA procedure used for the experimental data to these simulated population activities to test the reliability of our peak delay estimation (S18K Fig). Across all tested conditions, the CCV peak closely matched the imposed ground-truth delay, demonstrating that the estimator reliably recovers the direction and magnitude of inter-population temporal offsets rather than producing spurious short positive delays. These results support the interpretation that the positive delays observed in the experimental data reflect genuine delayed interactions captured by the analysis framework.

We additionally tested a range of parameters to check the robustness of the estimated delays per session, using CCA analysis. These include all possible combinations of; bin size (1, 5, or 10 ms), window length (10, 30, or 50 ms), step size (10, 30, or 50), and jitter window (10, 30, or 100 ms). We first applied CCA using all combinations of parameters on ground truth data (Poisson simulation for 300 trials) with +4 ms delay. Peak delay (circles in S19A Fig) showed perfect consistency across parameters (+4 ms) with a bin size of 1 ms. As expected, with a bin size of 5 ms, the peak moved to +5 ms and with a bin size of 10 ms, it was either at 0 or +10 ms (S19A Fig, middle and bottom). Next, we extended the analysis over parameters to an example session (0–100 ms from air puff and ± 50 ms delays) and found similar consistency of the peak delay across most of the parameter combinations (S19B Fig). A window size of 30 ms, together with finer steps, produced a stable delay estimation in the example session. Interestingly, jitter window did not affect the stability of the delay estimation, indicating that the relevant spike timing alignment between S1 and M2 underlying the communication subspace occurs at timescales substantially shorter than jitter windows tested (<10 ms). We applied time-shifted CCA across all sessions and parameter combinations and obtained uncorrected and jitter-corrected CCV peak delays (S20A and S20B Fig). This extensive analysis revealed the stability of the peak CCV timing across combinations of parameters. For a 1 ms bin-size, the distributions of peak delays across parameters remained significantly shifted toward positive values within ±10 ms range in both uncorrected and jitter-corrected analyses (S20C Fig, top row; uncorrected average across sessions = +1.7 ms, *p* = 0.004; jitter-corrected average = +1.1 ms, *p* = 0.021; *n* = 50 sessions, one-sided mouse-level sign-flip permutation tests; see Methods). With a larger 5-ms bin-size, the distributions similarly showed a positive bias, although the estimates became discretized at 0 or +5 ms due to the coarser temporal resolution (S20C Fig bottom row). Furthermore, this positive-delay bias in the distributions was more prominent in high-selectivity sessions compared to low-selectivity sessions (S20D Fig; high-selectivity: uncorrected average = +3.1 ms, *p* = 0, jitter-corrected average = +2.0 ms, *p* = 0.010, *n* = 25 sessions; low-selectivity: uncorrected average = +0.26 ms, *p* = 0.38, jitter-corrected average = +0.25 ms, *p* = 0.35, n = 25 sessions, one-sided mouse-level sign-flip permutation tests), consistent with the main findings. These results show that the central conclusion—a small positive coupling latency consistent with rapid feedforward communication from S1 to M2—is robust across a broad range of reasonable analysis parameters.

Furthermore, we applied two alternative directed measures of inter-areal interactions to consolidate these results: (1) pairwise cross-correlation analysis and (2) generalized linear modeling approach (GLM).

First, we investigated cross-correlations between the spiking activity of the two areas. We selected spikes within 100 ms from the air puff. Plotting the cross-correlogram (CCG) in an example session revealed that a large proportion of pairs had a small positive delay (S1 unit leading M2 unit) compared to their shuffled CCGs (where the CCG matrix elements were randomly resampled across delays and units) (S21A Fig). Jitter-corrected CCGs [76] (with 50 ms jittering window) showed a similar, high proportion of short-positive delays compared to the shuffled data (S21B Fig). Pooling all the pairs across sessions, peak delay histograms revealed a prominent number of pairs with a short positive peak delay in both raw and jitter-corrected CCGs across all sessions compared to the peak delay of the shuffled data (S21C Fig: raw average peak delay = +1.5 ms, *p* = 0.002; shuffled average peak delay = −0.07 ms, *p* = 0.8; S21D Fig: jitter-corrected average peak delay = +1.0 ms, *p* = 0.003; shuffled average peak delay = +0.05 ms, *p* = 0.1; *n* = 25 high-selectivity sessions, one-sided mouse-level sign-flip permutation tests on data within ±10 ms). The distribution of the peaks closely matches those of CCA analysis (S17D Fig) and reinforces the idea that the two areas are coupled in a predominantly feedforward fashion compatible with a direct pathway. To further illustrate the direction of coupling between strongly connected pairs we filtered all pairs across sessions based on the strength of correlation (peak CCG > 3.29 × standard deviation). The peak delay of most well-connected pairs was at a short positive delay (S21E Fig: average raw peak delay = +2.2 ms, *p* = 0.002; shuffled average peak delay = −0.14, *p* = 0.84; S21F Fig: average jitter-corrected peak delay = +1.8 ms, *p* = 0.002; shuffled = −0.009, *p* = 0.47; *n* = 25 sessions, one-sided mouse-level sign-flip permutation tests on data within ±10 ms).

GLM has been widely used as an alternative technique to analyze inter-areal coupling [40]. We therefore applied the same core principle to test interaction directionality in our dataset. We first tested the reliability of delay estimation using a ground truth simulation based on the previously described Poisson model. We ran the simulation for three predefined delays (−4, 0, and +4 ms). We then applied standard GLM to compute average weights of source neurons onto each target neuron across a range of delays (−20 to +20 ms). As shown in S22A Fig, the peak of average weights onto target neurons reliably recovered all three ground-truth coupling delays simultaneously.

Next, we applied this approach to our experimental data and revealed positive peak interaction delays across target (M2) neurons (S22B Fig). We averaged the regression weights across source neurons onto each target neuron for each delay as a proxy of population-level interactions at each delay. The median absolute peak delay across sessions was 4 ms (S22C Fig), matching the CCA-based delay estimator. Notably, the peak delay of individual target neurons was highly dependent on the session’s S1 selectivity (*p* = 8.3 × 10^−9^, *t* = 5.8, linear mixed-effects, *n* = 1,953 neurons with at least one non-zero weight across delays were included), with positive peak delays preferentially observed in high-selectivity sessions (S22D Fig), consistent with the CCA analysis (Fig 5D). Importantly, neurons with zero weights in the lasso model were not uniformly distributed across sessions. Instead, the fraction of zero-weight neurons per session was strongly negatively correlated with the session’s S1 selectivity (*r* = −0.688, *p* = 3.5 × 10^−8^, *n* = 50 sessions, Pearson’s correlation), and most zero-weight neurons (~86%) originated from low-selectivity sessions.

GLM is commonly applied to neuronal data using Poisson distributions to account for the statistical properties of the data. Using the Poisson-GLM approach, we found results consistent with those from the standard GLM described above. The median absolute peak delay across sessions was 4 ms, and the positive peak delay of each target neuron was significantly correlated with session’s S1 selectivity (*p* = 6.6 × 10^−6^, *t* = 4.5, linear mixed-effects, *n* = 2,917 neurons with non-zero weights were included; 111 neurons with zero weights were excluded, all from low-selectivity sessions), consistent with the results obtained using the standard linear model (LM) (S22E Fig). Therefore, our estimation of the coupling delay between S1 and M2 is reproducible via alternative analyses such as GLM-based coupling.

Finally, we considered the potential effect of unit-isolation quality on our delay estimations. Using ~80% of high-quality units with a low ISI violation rate (Methods, S23A Fig), we found consistent results regarding the short positive delay of the CCV across sessions (S23B–S23E Fig, median = +3). We consistently found significant effect of S1 selectivity (*S1sel*) and dimension (*dim*) on the absolute peak delay using either all the units (S17G and S17J Fig) or high-quality fraction of the units (S23F–S23H Fig, *p*_*dim*_ = 0.007, *t*_*dim*_ = −2.7, *p*_*S1sel*_ = 0.006, *t*_*S1sel*_ = −2.8, *p*_dim × S1sel_ = 0.38, *t*_dim × S1sel_ = 0.88, *df* = 392, linear mixed-effects). Consistently, we found a small absolute peak delay (median = 5 ms) using the first canonical dimension in the high-selectivity sessions even after jitter correction. These analyses demonstrate that the observed coupling structure and short positive delays are robust to stricter unit-quality criteria and are not attributable to multiunit contamination or spike-sorting artifacts.

These complementary analyses show that CCA-based estimation of the coupling delay between S1 and M2 is reproducible via alternative analyses such as GLM-based coupling and pairwise cross-correlations. Overall, we found delays consistent with the range (5–10 ms) of reported latencies between mouse sensory and motor cortices [74,75,77]. However, establishing these short delays as direct interactions by ruling out potential common input requires more targeted experiments.

Altogether, we revealed a transient, selective, and feedforward-biased communication between S1 and M2 that predicts behavioral performance. This rapid interaction was temporally locked to stimulus onset and contained task-relevant sensory coding information. These findings suggest that S1 sends selective task-relevant information to M2 shortly after stimulus onset to guide behavioral choice (go versus no-go). Thus, our findings uncover a robust feedforward functional connectivity motif linking somatosensory and premotor cortices that underlies successful sensorimotor transformation.

## Discussion

Taking advantage of simultaneous multichannel electrophysiological recordings during a delayed tactile discrimination task, we examined moment-to-moment interactions between S1 and M2 at single-neuron resolution. We identified a transient enhancement of short-latency feedforward interaction from S1 to M2 that emerged shortly after the stimulus presentation and subsided quickly, consistent with a fast feedforward communication pathway between the two regions. The strength of this transient interaction predicted behavioral performance, and its underlying pattern of activity was aligned with the stimulus-coding subspace, indicating its role in transmitting task-relevant sensory information to frontal premotor circuits. In contrast, feedback interactions were weak and reflected shared tuning properties between the two regions. Furthermore, brief optogenetic suppression of S1 demonstrated that early S1 activity is required for both successful performance in the go/no-go task and the emergence of robust stimulus selectivity in M2, consistent with a functional contribution of the transient S1–M2 coupling to the behavioral performance. These results illustrate dynamic, transient, and behaviorally relevant functional interactions between somatosensory and premotor cortical areas during perceptual decision-making.

In our task design, go versus no-go trial types are consistently linked to stimulus laterality. Consequently, our design does not permit a complete separation of decision-related processes from lateralized sensory processing. Importantly, however, this limitation does not alter our primary conclusions. Our goal was not to identify neural signals that distinguish neural processing during go versus no-go trials, but rather to characterize the temporal organization and functional significance of communication between S1 and M2 during sensorimotor transformation. The principal findings—including the identification of a rapid, transient feedforward S1–M2 communication shortly after stimulus onset, its relationship with behavioral performance, the alignment of the communication subspace with stimulus-coding subspaces, and its causal contribution to early M2 selectivity and behavioral discriminability—are all derived from the dynamics of contralateral S1–M2 communication itself, rather than from comparisons between go and no-go trials. These results therefore establish the existence and functional importance of a fast feedforward-biased sensorimotor communication channel, irrespective of whether the underlying computations reflect sensory lateralization, decision-related processing, or a combination of both. Determining the relative contributions of sensory laterality and decision-related processing will require behavioral paradigms in which stimulus laterality and behavioral choice can be manipulated independently, together with simultaneous recordings from both hemispheres.

We analyzed S1–M2 interactions in terms of total and noise correlations. Total correlations represent all possible interactions that arise from joint response to task variables. Importantly, when data are aligned to air puff onset, the joint response represents shared tuning properties of the two regions as well as trial-to-trial co-fluctuations in response (i.e., signal plus noise correlations [41,70]). To isolate co-fluctuations independent of stimulus-driven responses, we subtracted each neuron’s mean response across trials, yielding noise correlations [43]. As such, noise correlations can reflect the strength of moment-to-moment co-fluctuations regardless of the sensory tuning properties of the two regions. We analyzed both total and noise correlations to assess the nature of interareal co-fluctuations and their behavioral relevance. The timing and subspace of these interactions were consistent across both measures in the feedforward direction. However, comparing total and noise interactions in the feedback direction revealed a weaker interaction in the signal domain. Therefore, our results suggest that tuning properties in M2 are shaped by a strong feedforward signal from S1, and M2 in turn can influence late activity in S1. Notably, feedforward interactions occurred with small delays consistent with direct coupling observed between S1 and M2 [74,75,77]. Overall, both measures of coupling (total and noise) consistently revealed a rapid and transient feedforward interaction between S1 and M2 immediately following air puff presentation. This early coupling predicted behavioral performance and was highly dependent on the stimulus selectivity of the S1 population. Beyond this initial 100 ms after stimulus onset, when stimulus-evoked responses produced higher CCV_total_ than CCV_noise_, the two measures became almost identical and no longer exhibited behaviorally relevant dynamics.

How does M2 use feedforward information shared with S1? Previous studies have identified M2 as an important cortical region linking sensory information to decision-related processes [78]. It has a causal role in whisker-based goal-directed task performance [11,12] and contains neurons encoding both sensory [12,23,79] and choice- and action-related signals [12,80–84]. Compared to other frontal areas, rodent M2 is strongly connected with sensory areas [49,85,86], and its sensory signals are thought to originate primarily from the secondary somatosensory cortex (S2) [12]. It is known that S2 and other associative cortical regions send task-relevant feedback that dynamically interacts with S1 during behavior [21,87–89] and potentially contribute to shaping choice-related activity in M2 and other frontal areas. Our results extend this framework by showing that S1 potentially contributes to the prompt delivery of reliable sensory information to M2. This rapid transmission likely supports the timely coordination of downstream choice-related circuits essential for decision-making and learning.

Feedback from higher-order brain areas such as M2 to primary sensory regions is generally thought to support perceptual task performance. Previous studies established a functionally effective feedback circuit from M2 to S1 [22,73]. Primary sensory areas also appear to interact with the associated higher-order areas during spontaneous activity in a feedback direction [43]. In contrast, our results suggest a persistent feedforward interaction even during the inter-trial intervals. In line with recent findings [90], these persistent feedforward interactions may help to configure task-relevant preparatory states in the frontal motor areas. While strong feedback can be crucial for contextual suppression [56] or memory consolidation [73], it may be less essential during tasks with strong and unambiguous sensory evidence. In such cases, the key challenge of the nervous system is to reliably and efficiently transform sensory information into appropriate actions. Our results suggest that during skilled performance guided by clear sensory cues, animals engage distant sensorimotor circuits briefly and quickly during the initial sensory representations, with little subsequent coupling, which implies minimal interference between sensory-related neuronal activity and early choice-related activity in M2. This pattern is also compatible with the efficient coding hypothesis [91,92] where only behaviorally relevant information is transmitted along a hierarchy of brain networks. Consistent with this framework, our results suggest that rapid and transient feedforward communication of local sensory codes can further reduce the computational cost of perceptual decision-making. Nevertheless, more persistent inter-areal communication may be required during learning or when animals perform more challenging or ambiguous tasks.

Our task design did not force mice to withhold liking during the delay period, resulting in substantial variability in reaction times across animals. Yet we identified a subset of sessions in which the median reaction time exceeded the combined duration of the air puff and delay periods (>1.2 s, 36 sessions from 16 mice), allowing us to examine inter-areal interactions during the delay. In these sessions, we found no significant rise in S1–M2 coupling, and neither session-level nor trial-level coupling predicted behavioral performance. Furthermore, the communication subspaces underlying these weak interactions were not aligned with the choice-coding subspaces in either area. Nevertheless, we cannot rule out the possibility that nonlinear or recurrent inter-areal communication emerges during the delay period under different behavioral conditions, such as tasks with longer delays, distractors presented during the delay period, or paradigms that explicitly require sustained memory maintenance.

A prominent feature of the brief interaction was its strong feedforward bias. Previous studies have emphasized feedback projections to S1 from S2, M2, and M1 [20–22,73,87,93], and numerous studies have highlighted the role of M2 in the context of movement control [94], predictive coding [79,95,96], flexible switching [80], skilled behavior [97], and action suppression [98–100]. Across these lines of work, M2 is often portrayed as a leading controller of behavior that provides feedback to sensory areas. Our results complement this view by identifying when and how early sensory signals reach M2. Specifically, we show that task-relevant sensory information is transmitted to M2, likely via S1 through brief, precisely timed communication immediately after stimulus onset during perceptual decision-making. Quick feedforward broadcasting of sensory information to downstream cortical regions such as S2 and M2 may ensure that each area receives a reliable copy of the task-relevant sensory information needed for coordinated decision-making and subsequent feedback computations. In the whisker system, such feedforward signals may help premotor circuits to distinguish task-relevant sensory activity from activity generated by reflexive or voluntary whisker movements [17,65,101,102], thereby supporting accurate and timely sensorimotor transformations. Nevertheless, establishing direct contribution of S1 to these feedforward-biased interactions requires more targeted future experiments.

The barrel field comprises a large, highly organized portion of mouse S1 [49,93,103,104]. In our experiments, we delivered air-puff stimulation to the bulk of whiskers while recording from a range of anterior–posterior and medio-lateral coordinates in S1. Across sessions, the fraction of stimulus-selective neurons varied substantially, providing a natural gradient of population selectivity. We took advantage of this variation to demonstrate that the delay of peak interactions between S1 and M2 critically depends on the degree of stimulus selectivity in S1. This finding indicates that sensory information encoded by selective S1 neurons preferentially and rapidly aligns with the activity in M2 within a few milliseconds. Moreover, this relationship helps rule out non-specific network interactions between S1 and M2 as explanations for the observed coupling, such as within-modality spread of non-selective activity or global changes in cortical state induced by the stimulus presentation [60,61].

A previous study showed that inhibition of either S1 or M2 during stimulus presentation impairs performance in a similar delayed go/no-go task [12], indicating that both areas are required for correct performance in this task. Our results refine this understanding by demonstrating that transient coordination of activity between S1 and M2 reflects transferring of sensory information to the frontal cortex for triggering action-related neural dynamics in downstream areas such as ALM and M1 [12,105–107]. The identified S1–M2 communication channel is both selective and transient, carrying task-relevant sensory information only shortly after the stimulus presentation. This precise timing provides a temporal framework for future studies aimed at dissecting sensory, mnemonic, and premotor signals in M2 and prefrontal areas.

Recent advances in multivariate analyses of spiking activity [39,40] have enabled investigation of interareal communication at single-cell resolution, between sensory and association areas [43,44,48,56], between primary and secondary motor areas [108], and across distributed multi-region networks [109]. By combining multichannel silicon probe recordings with a time-shifted multivariate analysis, we identified the fine temporal structure of interareal interactions during a perceptual decision-making task. We revealed that shared activity patterns between the two regions are aligned with stimulus-coding subspaces, consistent with recent reports of learning-dependent activity alignment between S1 and posterior parietal cortex [44,56]. Studies in the mouse frontal cortex including the M2 area indicate that stimulus selectivity emerges with perceptual learning [12,23]. Our results suggest that early M2 selectivity emerges, at least in part, from interactions with the primary sensory cortex. This framework provides a basis for future investigations into how S1–M2 coupling evolves during learning or declines with aging, in which M2 population selectivity is known to sharply decrease [110,111]. Another promising direction is to investigate the role of sensory association areas in shaping M2 selectivity, especially under more demanding task conditions such as reversed or context-dependent reward contingencies.

Overall, our study reveals that communication between S1 and M2 during simple perceptual decision-making is brief, feedforward-biased, and occurs in a subspace aligned with the stimulus-coding directions of each area. Strikingly, we did not find strong feedback-biased interactions, in contrast to previous studies on primary sensory and sensory association areas [43,56]. This suggests that clear stimulus-response relationships may not require robust frontal feedback, or that such feedback may be relayed via other cortical pathways rather than directly from M2. These findings refine our knowledge of sensorimotor transformation and highlight the importance of transient, selective, and feedforward interareal communication in supporting goal-directed behavior.

## Methods

### Ethics statement

Experimental procedures were all approved by the Animal Care and Use Committee (IACUC) at Nanyang Technological University (AUP No. A19062 and A24054) and adhered to Singapore’s National Advisory Committee for Laboratory Animal Research (NACLAR) guidelines.

### Animals

Adult, male and female mice (Ai32, Jackson Lab stock 012569) were used for this study. Ai32 mice were crossed with PV-Cre mice (Jackson Laboratory stock 017320), and the offspring (PV-ChR2 mice) were used for optogenetic experiments. Animals were housed on a dark/light cycle of 12 h/12 h (light on from 7:00 to 19:00) at a temperature ~21 °C and humidity of 62%. Experiments were carried out in the light cycle between 9:00 and 18:00.

### Head-bar implantation and behavioral training

Mice were initially anesthetized with 3% isoflurane in oxygen in a chamber and continued with 1.5%–2.0% on a stereotaxic frame (RWD Life Sciences). Animals received 0.05 mg/kg buprenorphine subcutaneously before surgery and 5 mg/kg meloxicam after surgery. A custom-made head-bar was implanted behind and parallel to the lambdoid sutures on the skull using veterinary tissue glue (3M Vetbond) and black dental cement (Contemporary Ortho-Jet, Black, Lang Dental). Mice were returned to the home cage with free access to food and water and monitored for a week to recover from the surgery. After the recovery period, mice were water-restricted (1 ml per day) for 2 days and were habituated for 2–4 days to head-fixation before training in a delayed go/no-go task controlled by the Presentation software (Neurobehavioral Systems). Each trial began with a 200-ms air puff delivered onto the distal half of the long whiskers on either side of the head. An air puff was followed by a 1-s delay, and white noise was played via a speaker for a maximum of 2 s to cue the response window. We built an IR beam-breaker circuit using separate emitting and detecting LEDs. The LEDs were carefully positioned to register any licking behavior. Beam interruption by tongue was registered via a National Instruments board (USB-6001) and fed to Presentation software to control task events. In go trials, mice were required to lick during the auditory cue to receive a 2.3 μL drop of water via a metal lick port. Miss trials, defined by the absence of a lick during the response window, resulted in neither reward nor punishment. A lick response in no-go trials resulted in a 6-s time-out, added to the inter-trial interval (5.1–7.0 s). Mice typically performed around 300 trials. In most recording sessions, a maximum of 300 trials was predetermined. As a result, most of the sessions (76%) contained 300 trials and 90% contained between 250 and 400 trials.

### Craniotomy and electrophysiology

After reaching a stable daily performance, mice were anesthetized with 3% isoflurane in the chamber, followed by 1.5%–2.0% isoflurane on the stereotaxic frame. Two craniotomies (diameters up to ~1 mm) were drilled over the right hemisphere area corresponding to S1 (centered at AP: −1.7 to −2 from bregma and ML: 3.0 to 4.0 from midline) and M2 (centered at AP: 1.3 to 2.2 mm from bregma and 0.5 to 1 mm from midline). Craniotomy sites were sealed with a silicone elastomer (Kwik-Cast, World Precision Instruments). Electrophysiological recording began at least 4 hours after craniotomy to allow mice to recover from anesthesia. Two silicon probes (ASSY-37 H8b, Acute 32-channel H8b probe, 1 shank per recording area, 30 µm site spacing, 9 mm length, Cambridge NeuroTech) were coated on the back side with DiD stain (Invitrogen) and lowered into S1 and M2 using a pair of motorized micromanipulators (MM-500, RWD Life Science) independently at a depth range of 1,000–1,400 µm. Wideband neural signals were sampled at 30 kHz with RHX data acquisition software and Intan 128-channel acquisition system (M4200, Intan Technologies) during the task. Signals from task events and the IR beam-breaker circuit were recorded along with the electrophysiological data at sync. Kwik-Cast was reapplied after each daily recording session to seal the craniotomies. In subsequent recording sessions, silicon probes were inserted into a fresh site, at least 200 μm away from the previous sites. Recording sessions were performed in subsequent days. Each mouse was recorded for 1–4 sessions, with most animals undergoing 3 sessions of recording. After the final recording session, mice were deeply anesthetized and perfused with 0.1 M PBS, followed by 4% paraformaldehyde dissolved in PBS. Brain samples were removed and post-fixed overnight at 4°C, then transferred to 30% sucrose in PBS for 2–3 days at 4 °C. After embedding in OCT, brains were frozen and cut into 100 μm coronal sections using a Leica CM1950 cryostat. The slices were mounted on microscopic slides with Fluoroshield containing DAPI (Sigma-Aldrich) and were imaged with Axio Scan.Z1 microscope (ZEISS).

### Behavioral data analysis

Behavioral data were analyzed using custom-written MATLAB codes. Four trial types were defined based on their stimulus time and outcome; (1) hit trials when left air puff triggered licking response, (2) miss trials when mice failed to respond during the response window, (3) false alarm (FA) when mice responded incorrectly during the white noise presentation following a right air puff and (4) correct rejection (CR) when mice withheld licking response during the white noise presentation following a right air puff. Hit and CR rates were defined as the fraction of correct go and no-go trials, respectively. Behavioral performance (discriminability of the stimuli) was computed as d-prime:


$$\begin{array}{c} d^{\prime }=Z(Hit\;Rate)-Z(1-CR\;rate) \end{array}$$ (1)

where Z is the inverse of the cumulative distribution function of the standard normal distribution.

Stimulus and lick event data were analyzed in MATLAB using custom-written code to identify lick and stimulus onset times. Lick events were detected by thresholding digital signals with debouncing at 0.8 V and then removing subsequent high voltage values up to 50 ms, which ensured sufficient debouncing while staying below the licking cycle across all animals. A first-lick response was defined by setting a 1-s refractory period on lick events and selecting the first lick in each bout. For response alignment, first licks occurring within 0.2–6 s after a left air puff were selected. To isolate the epochs of poor engagement, a local hit rate was computed by fitting a local regression function (*smooth* function in MATLAB with *lowess* method and a span of 10 trials) on zero and one hit response vectors. A threshold of 0.2 on the local hit rate effectively isolated epochs of poor engagement.

### Electrophysiology data analysis

Neural data for each recording probe were separately analyzed for spike isolation using the HerdingSpikes algorithm [112] as part of SpikeInterface [113] in Python, and manually curated with Phy (https://github.com/cortex-lab/phy) as described previously [110]. Broadband data were filtered at 0.3–3 kHz and re-referenced before spike sorting. Spike clusters were manually curated to exclude noise and further isolate spikes based on features provided by Phy’s graphical user interface. These features included amplitude, ISI distributions, correlograms, firing rates, individual and average spike waveforms, and cluster localizations on the shank. Whenever required, clusters were separated from each other based on amplitude drift patterns and ISI distributions, suggesting multi-unit activity. Two or more clusters were merged if the spike localization, correlograms, and amplitude drift patterns suggested that they belonged to the same neuron. All valid spike clusters were included for further analyses regardless of single-unit isolation quality. To test the potential effect of single-unit isolation quality on the results, we computed an ISI violation rate defined as the fraction of ISIs smaller than 2 ms among all ISIs smaller than 200 ms. By thresholding the ISI violation rate at 0.01 in M2 and 0.04 in S1, we included ~80% of units with low ISI violation rates in each session. Average firing rates for the example sessions were calculated using 1-ms binning and 5-ms smoothing (moving average). Baseline firing rate (0–15 ms from air puff) was subtracted from each neuron’s average firing rate.

### Optogenetic suppression of S1

PV-ChR2 mice, in which parvalbumin-positive inhibitory neurons express channelrhodopsin-2, were used for optogenetic suppression of the S1 using a fiber-optic-coupled 473 nm blue laser (Shanghai Laser & Optics Century Co.). The open end of the fiber optic was fitted with a zirconia ferrule, and an aluminum foil sleeve was custom fitted onto the ferrule to minimize exposure to scattered laser. During head-bar implantation surgery, the skull above whisker S1 on both hemispheres was thinned and a layer of UV-curing adhesive (Norland Optical) was applied to the area after a thin layer of 3M’s Vetbond to gain optical access into the cortex [12]. To minimize scattered laser light reaching the animals’ eyes, the exposed skull was surrounded by a high wall (4–6 mm high) made of dark acrylic cement. Mice were trained on the go/no-go task previously described. After they reached 70% correct rate for 3 consecutive days, a control experiment was performed by shining a spot of laser (1.5 mm in diameter and a power density of 6.4 mWmm^−2^) on a piece of aluminum foil that was covering the whole skull. The spot was positioned at the right S1 coordinates by attaching the fiber to a motorized micromanipulator. Laser was applied continuously from 100 ms before to 100 ms after the air puff onset in every one out of one-third of the go and one-third of the no/go trials. An additional 100 ms linear ramp-down to zero was applied to prevent the rebound activity in excitatory neurons. Trials were pseudo-randomly interleaved in a manner that only allowed 2 subsequent laser trials with at least a 5.3-s interval between subsequent laser onsets in such trials. Following the control experiment, the laser was delivered to the right S1 (centered at −1.7 mm from bregma and 3.5 mm from the midline) and then to the left S1 in subsequent sessions using the same mice.

### Selectivity index

To compute the selectivity index (SI), peri-stimulus spikes of each neuron were binned using 100-ms intervals. A Receiver Operating Characteristic (ROC) analysis was performed for multiple 100-ms windows relative to the time of air puff using spike counts of go trials as “positive signal” and those of no-go trials as “negative signal” distributions. By calculating true positive rates and false positive rates at multiple thresholds, an ROC curve was constructed. Area under ROC curve (AUC) was computed with the trapezoid technique (trapz in MATLAB). AUC represents the difference in spiking activity between left and right stimuli, considering trial-to-trial variability in firing rate. AUC was scaled between −1 and 1 to represent SI:


$$\begin{array}{c} SI=2\times (AUC-0.5) \end{array}$$ (2)

To test statistical significance, we used a resampling technique. Two shuffled spike count vectors were constructed by randomly resampling from the pool of spike counts of all trials. SI was recalculated based on these resampled data and the process was repeated 250 times. The resulting SIs formed a null distribution. A *p*-value was computed by thresholding the null distribution at the value of the original SI. Sessions were divided into two groups of selectivity based on the significance of SI (*p* < 0.05, without correcting for multiple comparisons) in the first post-stimulus time window (0–100 ms). Sessions were classified as “high-selectivity” if they were above the median for the fraction of selective S1 neurons (median = 29%). This measure was highly correlated with the average selectivity of all S1 neurons in each session (*r* = 0.95, *p* = 2.06 × 10^−26^, Pearson’s correlation, *n* = 50 sessions) indicating that the fraction of selective neurons is a reliable proxy for the overall sensory selectivity of the S1 population. Most downstream statistical analyses use population selectivity as a continuous variable rather than a thresholded categorical grouping (e.g., linear mixed modeling relating S1–M2 coupling to behavioral performance).

### Canonical correlation analysis

Population activity of neurons in each area evolves in a multi-dimensional space where each neuron represents one dimension. Canonical correlation analysis (CCA) relates the spiking activity of two neuronal populations by finding latent dimensions (canonical pairs) representing shared patterns of activity between two regions [39]. The first canonical pair represents the activity along a one-dimensional subspace that captures maximum correlation between the two areas and subsequent canonical pairs capture weaker correlations along independent subspaces. The objective of CCA is to find axes a and b along which the activity is maximally correlated between the two regions:


$$\begin{array}{c} \underset{a,b}{arg\;max\;}corr(Xa,Yb) \end{array}$$ (3)

where X is n×p and Y is n×q matrices of activity where n denotes samples. p and q denote the number of dimensions (e.g., neurons) in X and Y, respectively. a and b are 1×d vectors where d is the minimum of p and q. These vectors can be thought of as dimensions or axes in the neuronal activity space denoted as ρd, along which the two areas interact. Canonical correlation value (0 ≤ CCV ≤ 1) captures the magnitude of correlation between Xa and Yb. In practice, CCA returns matrices A and B where columns of each matrix are independent axes we term “canonical dimensions”. These dimensions are sorted by the magnitude of correlation along each axis. For all analyses, we used the first canonical dimension unless explicitly stated.

We used a time-shifted CCA [43], where for each time window in X an equal but shifted time window of Y was used as input to CCA. In each CCA iteration, we computed up to 4 canonical dimensions. Data was prepared for CCA analysis by isolating spike times of each unit in each trial before and after the first lick or stimulus. Event-aligned spike trains were then binned in each trial using 5-ms intervals. For each iteration of CCA, we used a 50-ms time window consisting of 10 spike count bins, moving with 10-ms steps. Because the CCA algorithm requires the inversion of the covariance matrices [114], neurons with no spikes within the analysis window across trials should be excluded. After this filtering, most sessions (~70%) contained at least 50 neurons in each area (S3H Fig). The activity of each neuron was then z-scored for all CCA analyses. To isolate moment-to-moment co-fluctuations (i.e., “noise correlations”), trial-averaged activity of each neuron was subtracted from the z-scored activity of each trial. For each time window, a $t_{Y}$ was chosen at a delay of $t_{Y}-t_{X}$, where t is the first sample in the 50-ms chunks of the data. We moved ${\text{t}}_{𝐘}$ in either direction, one sample (5 ms bin) at a time, to obtain shifted iterations of the data. We assigned S1 activity to X and M2 activity to Y. Therefore, the sign of delay determines the direction of interactions; a feedforward interaction (S1 → M2) should occur in positive delays and a feedback interaction (M2 → S1) in negative delays. To evaluate temporal dynamics of interactions in each session, CCVs (Fig 2C–2H) were averaged across 5 delays {−10 ms, −5 ms, 0, +5 ms, +10 ms}. This yielded a CCV vector showing peri-stimulus dynamics of CCV for each session. To test significance of the fluctuations relative to the air puff onset, a 95% confidence interval for the mean of baseline CCV across sessions (CCV CI) was computed by first taking CCV from −0.25 s to 0 relative to the air puff onset, yielding a $N_{session}\times T_{baseline}$matrix. The CI was computed using bootci function in MATLAB (R2019b) with the following parameters; nboot: 1000, bootfun:@mean, type: 'bca' and alpha:0.05 as well as the input data matrix ($N_{session}\times T_{baseline})$. The output was a $\text{2}\times T_{baseline\;}$matrix for upper and lower confidence bounds for which the mean values were used to assess deviations from baseline. For response-aligned data, the baseline CCV was taken from −3 s to −2 s relative to the first lick onset. Time-averaged values of CI crossing points in each side of CI were compared against the time-averaged values of the baseline CCVs to verify significant deviations from the baseline using Wilcoxon signed-rank tests. A shuffled CCV was computed for each session using the same procedure as the original CCV, except using trial-shuffled S1 activity to remove the trial-to-trial correspondence between S1 and M2 activity while preserving the temporal structure of the activity in each area. To obtain a higher temporal resolution, spike trains were binned within 1-ms intervals, resulting in 50 samples for each iteration of CCA. This 50-ms window was moved without overlapping (with a step of 50 ms) and each delay step was 1 ms. Corrected CCV maps were computed by subtracting jittered-CCV to reveal potential direct interactions [43]. Jittered-CCV was computed by randomly resampling spikes within 25-ms windows across trials [115].

### Geometric mean firing rate

The geometric mean firing rate (gmFR) of each area was computed by averaging across trials and neurons within each 50-ms window, in parallel with CCA analysis for the same period of activity in both regions:


$$\begin{array}{c} gmFR=\sqrt{\mu_{x}\times \mu_{y}} \end{array}$$ (4)

where $\mu_{x}$ and $\mu_{y}$ are grand average spike counts of X and Y, respectively in a 50-ms window. gmFR was also calculated for delayed iterations, yielding the same temporal map as with the CCV. In parallel with CCV, to evaluate the temporal dynamics of firing rate in each session, gmFR was averaged across 5 delays {−10 ms, −5 ms, 0 , +5 ms, +10 ms}.

### Coding direction

Coding direction (cd) is an axis that maximally separates evoked activity of left and right air puffs and can be computed by fitting a LM (i.e., a linear classifier). Due to the high dimensionality of the population activity, a limited number of trials and often high trial-to-trial variability in response, linear models such as linear discriminant analysis or support vector machines can overfit the data and distort the actual coding direction. A simpler LM is advisable in this situation. Therefore, we computed coding direction (cd) based on a procedure frequently used in the literature [68,69]. In each area, average spike counts within 100 ms from left air puff onset were subtracted from those from corresponding spike counts evoked by right air puffs:


$$\begin{array}{c} cd=\;{\overset{―}{r}}_{left}-{\overset{―}{r}}_{right} \end{array}$$ (5)

Each element of the vector $\overset{―}{r}$ was the trial-average spike count for each neuron in the population. To evaluate the consistency of cd over time, we computed a cd for multiple 50-ms windows relative to the stimulus and then constructed a correlation matrix using Pearson’s correlation coefficients. High correlation between cd in each window with a subsequent window indicates that the same pattern of activity is sustained, and a low correlation indicates a dissimilar activity pattern.

### Similarity between cd and ρd

We computed the absolute value of the cosine similarity between cd and ρd which represents the alignment of a stimulus coding subspace with an interaction subspace:


$$\begin{array}{c} similarity=|cos(\theta )|=|(\frac{cd\cdot \rho d}{∥cd∥.∥\rho d∥})| \end{array}$$ (6)

We took the absolute value of the cosine similarity as the magnitude of alignment between cd and ρd since the direction of ρd may flip towards −cd due to the delayed *trans-*callosal arrival of a right-selective activity. With this assumption, we generalize the similarity between the two vectors to both cases of selectivity. To compute cosine similarity based on independent trials (i.e., cross-validation), trials of each session were randomly split into two; one half was used to estimate the local coding axis, and the other half for the communication axis. Similarities were then computed as in equation (6) using temporally independent cd and ρd for each session.

### Trial-level coupling

To quantify trial-level coupling, we applied CCA analysis on residual activity (for noise correlations) using 2-ms bins and a 100-ms sliding window (10-ms steps), which yielded 50 temporal samples for each trial. All trials within each session were used to obtain a reliable estimate of the first pair of canonical weights (a and b) and projected all trials along the first canonical dimension (Xa and Yb). For each window and delay combination, a Pearson’s correlation coefficient was calculated between the projected population activity of S1 and M2 in each trial. These correlation coefficients represent the within-trial coupling strength between the two areas. Within-trial coupling strength was estimated as the average of these correlation coefficients within 100 ms of the air puff in either the feedforward (FF: 2–20 ms) or feedback (FB: −20 to −2 ms) direction.

As a complementary analysis, we repeated the trial-level coupling using 10-fold cross-validation. For each fold, the first pair of canonical weights was estimated from 90% of the trials (training trials) and applied to the remaining 10% of held-out trials (test trials). Test-trial activity was projected onto the canonical dimensions obtained from the training trials ($X_{test}a_{train}$ and $Y_{test}b_{train}$) and within-trial coupling was calculated from the Pearson’s correlation coefficient between the projected S1 and M2 activity.

### Simulation of interareal interactions

To simulate interareal interactions, we modeled spiking activity of two populations with a simple Poisson process. For neuron n in trial m, the spike probability was defined as


$$\begin{array}{c} P\left({spike}_{n,m}\right)=\lambda_{n,m}(t)dt \end{array}$$ (7)

where λ is the sum of three components: (1) a baseline firing rate of 5 Hz, (2) a trial-to-trial fluctuation randomly sampled between 0 and 10 Hz for each trial, and (3) a coupling term. For population 1, the coupling term was set to 0, implementing negligible feedback coupling from population 2 to population 1. For population 2, the coupling term was defined as 0.2 times the summed spiking activity of population 1 at a given time point, shifted by the ground-truth delay, thereby implementing feedforward coupling from population 1 to population 2. During a predefined 50-ms interaction period (green shading S18I and S18J Fig), the coupling strength between the two populations was transiently increased 50-fold, where the coupling term for population 2 increased to 10 times the summed spiking activity of population 1, while the coupling term for population 1 remained 0. The ground-truth temporal offset between the populations was systematically varied from −10 ms (population 1 lagging population 2) to +10 ms (population 1 leading population 2). We repeated the simulation for 150 trials and for 70 neurons for each population.

For GLM-based peak delay estimation, we used a variation of the same Poisson model. In each of 300 simulated trials, a 50-ms coupling window was assigned and population activity in each 1-ms bin was defined as the sum of a 5 Hz baseline, 0–10 Hz trial-to-trial fluctuation (shared between populations) and a coupling term. For population 1, the coupling term was set to 0, whereas for population 2, it was set to 70 times the summed spiking activity of population 1 shifted by ground-truth delay. We ran the simulation for three predefined delays (−4, 0, and +4 ms, 20 neurons per population) and then concatenated neurons before applying the *lasso* regression across 21 delays (−20 to +20 ms).

### Pairwise cross-correlation analysis

A cross-correlation analysis was conducted between pairs of higher-quality S1 and M2 neurons (~80% in each area with a lower ISI violation rate as described above). Spikes within 100 ms of the air puff were binned in 1 ms intervals and concatenated across trials. CCG for neuron pair j,k was computed over M trials as [76,116];


$$\begin{array}{c} {CCG(\tau )}_{j,k}=\frac{\frac{\text{1}}{M}\sum_{i=\text{1}}^{M}\sum_{t=\text{1}}^{N-\tau }x_{j}^{i}(t)x_{k}^{i}(t+\tau )}{\theta (\tau )\sqrt{\lambda_{j}\lambda_{k}}} \end{array}$$ (8)

where N is the number of time bins, τ is the time delay between the spike train and $x_{j}^{i}(t)$ and $x_{k}^{i}(t+\tau )$ are spike counts in a time bin t of each trial i for S1 and a delayed time bin t+τ for M2 neurons. Normalizing denominators consist of a triangular function; θ(τ)=N−τ which corrects triangular decay over non-overlapping bins and geometric mean firing rate denoted by $\sqrt{\lambda_{j}\lambda_{k}}$ which corrects for CCG estimation errors caused by variable firing rates across pairs [58]. A jitter window of 50 ms was used to compute a jittered CCG for each pair [76,117]. CCGs were further corrected by subtracting this jittered CCG as follows;


$$\begin{array}{c} {CCG}_{jitter-corrected}=\;CCG-{CCG}_{jitter} \end{array}$$ (9)

Jitter correction accounts for slower firing co-fluctuations caused by stimulus- or behavior-locked activity and slow cortical oscillations which may not relate to direct communications. CCG was computed within ±50 ms delays and the peak delay was calculated after applying a 5 ms moving average and limiting CCG to ±40 ms.

### GLM-based peak delay estimations

To apply GLM to our inter-areal, we used *lasso* function in MATLAB, we systematically quantified neural coupling between S1 and M2 after z-scoring the activity of each neuron. Ten candidate values of the LASSO regularization parameter (*λ*) were evaluated, and 3-fold cross-validation was used to assess model performance across these values, with the *λ* yielding the lowest cross-validated deviance selected for the final model. The maximum number of optimization iterations was set to 10^5^ for *lasso*. The resulting model was used to predict each M2 target neuron from time-shifted activity of S1 source neurons. Each M2 neuron’s activity (0–50 ms of air puff, 25 bins of 2 ms × number of trials in each session) was regressed by its own history (−20 to −2 ms, thus 10 self-history predictors) and time-shifted activity of all S1 neurons (−20 to +20 ms, thus 21 × N_S1_ predictors), across all trials within each session. We averaged the regression weights across source (predictors) onto each target neuron for each delay as a proxy of population-level interactions. Neurons with zero weight across all delays were excluded from the subsequent statistical analysis testing the association between each target neuron’s peak delay and S1 selectivity. To ensure generalization of our results to the original spiking activity with its unique statistical properties, we repeated the analysis using *lassoglm* in MATLAB with a Poisson distribution (Poisson GLM, 10 *λ* with 3-fold cross-validation and 10^3^ as maximum iterations). Data parameters (e.g., temporal resolution was 2 ms) were identical to those chosen for the standard GLM described above.

### Statistics

All statistical analyses were performed using MATLAB and are described in the text and figure legends. All statistical tests were two-sided unless otherwise stated.

To test if variability in population selectivity is due to variable quality of the recordings, a linear mixed-effects model was fit to neuronal data with subject and session identifiers as random effects and the fraction of selective neurons in each area (Sel), as a fixed effects and baseline firing rate of each neuron as the response variable (BFR).


$$\begin{array}{c} BFR\;\sim \;Sel+(\text{1}|Subject)+\left(\text{1}|Session\right) \end{array}$$ (10)

Another linear mixed-effects model was constructed to test the behavioral relevance of CCV measures with behavioral d-prime as the response variable. Fixed effects included the ${S\text{1}}_{sel}$, fraction of selective M2 neurons (${M\text{2}}_{sel}$) and average CCV within 100 ms of air puff ($\overset{―}{CCV}$) with full interaction terms as:


$$\begin{array}{c} d^{\prime }\sim \;{S\text{1}}_{sel}+{M\text{2}}_{sel}+\overset{―}{CCV}+{S\text{1}}_{sel}:{M\text{2}}_{sel}+{S\text{1}}_{sel}:\overset{―}{CCV}+\;{M\text{2}}_{sel}:\overset{―}{CCV}+{S\text{1}}_{sel}:{M\text{2}}_{sel}:\overset{―}{CCV}+(\text{1}|Subject) \end{array}$$ (11)

To examine variables predicting delay of the CCV peak, the following linear mixed-effects model was used with Delay (delay of the peak of CCV), ${S\text{1}}_{sel}$(high or low selectivity), Correction (with or without jittered-CCV subtraction) and Dimension as fixed effects:


$$\begin{array}{cc} De & lay\;\sim \;{S\text{1}}_{sel}+Correction+Dimension\;+{S\text{1}}_{sel}:Correction\;+\\ & {S\text{1}}_{sel}:Dimension+Correction:Dimension+{S\text{1}}_{sel}:Correction:Dimension+(\text{1}|Subject) \end{array}$$ (12)

To test the effect of trial-level coupling on trial outcome, we applied a binomial mixed-effects model with trial outcome (correct versus incorrect) as the response variable, trial-level coupling as a fixed effect, and sessions and subjects as random effects:


$$\begin{array}{c} ogit\left(P(Correct)\right)\sim \;Coupling+\left(\text{1}|Subject\right)+\left(\text{1}|Session\right) \end{array}$$ (13)

To examine the possible association between the motion index (Motion) and trial outcome, we applied a binomial mixed-effects model with trial outcome as the response variable, trial-level motion index a fixed effect, and sessions and subjects as random effects:


$$\begin{array}{c} ogit\left(P(Correct)\right)\sim \;Motion+\left(\text{1}|Subject\right)+\left(\text{1}|Session\right) \end{array}$$ (14)

Potential interaction between trial-level motion index and coupling was examined in a binomial mixed-effects model as follows:


$$\begin{array}{c} ogit\left(P(Correct)\right)\sim \;Motion+Coupling+Motion:Coupling+\;\left(\text{1}|Subject\right)+\left(\text{1}|Session\right) \end{array}$$ (15)

To examine if the motion index can predict coupling at the trial level, the following linear mixed-effects model was used:


$$\begin{array}{c} Coupling\;\sim \;Motion+\;\left(\text{1}|Subject\right)+\left(\text{1}|Session\right) \end{array}$$ (16)

The association between GLM-based peak delay of individual source neurons and ${S\text{1}}_{sel}$ was tested using the following linear mixed-effects model:


$$\begin{array}{c} Delay\;\sim \;{S\text{1}}_{sel}+\;\left(\text{1}|Subject\right)+\left(\text{1}|Session\right) \end{array}$$ (17)

To assess the statistical significance of the positive peaks obtained across CCA parameter settings and across CCG pairs, we used a one-sided sign-flip permutation test at the mouse level. Session values were first averaged to obtain a single value for each session, and the grand average across all sessions was used as the observed statistic. For each of 1,000 permutations, an independent random sign (+1 or −1) was assigned to each mouse and applied to all session-wise average values from that mouse. The grand average of the sign-flipped session values across all sessions was then recalculated to generate a null distribution. The p-value was calculated as the proportion of permuted statistics that were greater than or equal to the observed statistic. This procedure tests the null hypothesis that the distribution of session-level values is symmetric around zero, while preserving the dependence among sessions from the same mouse.

## Supporting information

S1 Fig Behavioral performance of mice in the delayed go/no-go task. **(A–C)** Hit rate (A), Correct rejection (CR, B) rate and d-prime (*d*′, C) of 18 mice in the task (Mean ± SEM). **(D)** Performance of mice in 50 recording sessions. Color range indicates d-prime (*d*’) for each session. **(E and F)** DAPI (blue) staining marks cell nuclei and DiD (pink) stain marks probe tracks in S1 (E) and M2 (F). Scale bars: 1 mm. Overlayed gray outlines depict area outlines (modified from Allen Mouse Brain Atlas, P56 coronal sections, E: image 70 and F: image 34) [118].
(TIF)

S2 Fig Neuronal responses in two example sessions indicate reliable and selective responses in both regions. **(A)** Responses of simultaneously recorded neurons in S1 within 100 ms of air puff, sorted by depth of each neuron (rows), averaged over hit (left) and correct rejection (CR, right) trials. **(B)** Average firing rate of S1 across neurons in hit vs. CR (left) and hit vs. miss (right) trials (*n* = 73 neurons). **(C)** Responses of simultaneously recorded neurons in M2 in hit (left) and correct rejection (CR, right) trials. **(D)** Average firing rate of M2 across neurons in hit vs. CR (left) and hit vs. miss (right) trials (*n* = 95 neurons). **(E and F)** Quantification of S1 firing rates for Example session 2 (*n* = 129 neurons). **(G and H)** Quantification of M2 firing rates for Example session 2 (*n* = 114 neurons). Average plots are accompanied by the standard error of the mean (±SEM).
(TIF)

S3 Fig Firing rate and selectivity of neurons across 50 sessions. **(A)** Fraction of S1 and M2 neurons with significant initial selectivity (0–0.1 s from air puff onset) in each session (gray lines). The arrow points to the median (arrow) of the data in S1. The data underlying this panel can be found in https://doi.org/10.5281/zenodo.23338822 as S3A.xlsx. **(B)** Cumulative distribution of baseline firing rate (500 ms before stimulus) of all S1 (left) and M2 (right) neurons grouped based on the median fraction of selective S1 neurons. Population selectivity in neither region predicted the baseline firing rates of individual neurons in the same region (S1: *p* = 0.84, *t* = −0.2, *df* = 4,971, M2: *p* = 0.056, *t* = −1.91, *df* = 5,876, linear mixed-effects). **(C)** Grand average firing rate (in 100-ms bins) of S1 (left) and M2 (right) neurons across all trials in high selectivity (blue) and low selectivity (red) divisions. Black and gray arrows point to air puff and auditory response cue times, respectively. **(D)** Firing rate of S1 neurons averaged across all trials for high (left) and low selectivity (right) sessions. **(E)** Average firing rate of M2 neurons. **(F)** Left vs. right selectivity of S1 neurons in high (left) and low (right) selectivity divisions. **(G)** Left vs. right selectivity of S1 neurons in high (left) and low (right) selectivity divisions. Note that in F and G, neurons with no significant selectivity from 0 to 3s after air puff were excluded and time bins with a non-significant selectivity index are set to 0. **(H)** Distribution of the number of recorded neurons (left) and neurons included in the CCA analysis (right) per session in S1 and M2.
(TIF)

S4 Fig Temporal dynamics of interactions in trial types grouped by outcome and in response-aligned data. **(A–D)** Total interactions for each trial type; hit (A, *p*_up_ = 1.9 × 10^−5^, *z*_up_ = 4.3 and *p*_down_ = 1.6 × 10^−8^, *z*_down_ = −5.7, upper and lower CI crossing points respectively, within 1 s from air puff vs. baseline, Wilcoxon signed-rank test), correct rejection (CR, B, *p*_up_ = 0.00013, *z*_up_ = 3.8, *p*_down_ = 4.3 × 10^−8^, *z*_down_ = −5.5), miss (C, *p*_up_ = 0.0002, *z*_up_ = 3.7) and false alarm (FA, D) respectively. Geometric mean firing rates (gmFR, mean ± SEM) are plotted on the right y-axis in red. **(E–H)** Noise interactions in hit (E, *p*_down_ = 1.9 × 10^−8^, *z*_down_ = −5.6), correct rejection (F, *p*_down_ = 4.3 × 10^−8^, *z*_down_ = −5.5), miss (G, *p*_down_ = 6.8 × 10^−8^, *z*_down_ = −5.4), and false alarm (H), respectively. **(I, J)** Response-aligned data show decreases in total interactions in all sessions (I, *p*_down_ = 1.9 × 10^−9^, *z*_down_ = −6.0) and sessions with long median reaction time (mRT > 1.2 s, I, *p*_down_ = 5.4 × 10^−7^, *z*_down_ = −5.0). Lavender dashed lines represent 95% CI of the baseline CCV and the gray dotted line represents the average trial-shuffled data (one shuffle per session). The shaded area represents the standard error of the mean (SEM). Wilcoxon signed-rank test was used to test the differences between average upper and lower CI crossing points within 1s from the air puff onset vs. baseline (n = 50 sessions).
(TIF)

S5 Fig Distribution of trial-level coupling between S1 and M2 in correct and incorrect trials. **(A)** Probability density function (pdf) of trial-level coupling in the feedforward direction (FF, S1 → M2) for high selectivity sessions. Correct trials exhibited significantly stronger coupling than incorrect trials (*p* = 0.0045, *t = 2.84*, *df* = 5,242, linear mixed-effects, 25 sessions from 12 mice). **(B)** Distribution of trial-level coupling in the feedback direction (FB, M2 → S1) for high selectivity sessions with no significant difference between correct and incorrect trials (*p* = 0.24, *t* = 1.2, *df* = 5,242, linear mixed-effects, 25 sessions from 12 mice). **(C)** Distribution of trial-level coupling in the feedforward direction for low selectivity sessions with no significant difference between correct and incorrect trials (*p* = 0.80, *t* = −0.26, *df* = 5,065, linear mixed-effects, 25 sessions from 13 mice). **(D)** Distribution of trial-level coupling in the feedback direction for low selectivity sessions with no significant difference between correct and incorrect trials (FB: *p* = 0.53, *t* = 0.62, *df* = 5,065, 25 sessions from 13 mice.
(TIF)

S6 Fig Lack of association between motion index and the S1 → M2 coupling. **(A)** Mean motion index (±SEM) averaged across 25 high-selectivity sessions. The right axis (red) shows the average lick rate. **(B)** Trial-level measurement of z-scored motion index and S1 **→** M2 coupling show no significant correlation (*r* = −0.02, *p* = 0.145, 5,244 trials from 25 high-selectivity sessions, Pearson’s correlation). **(C)** Grand average trial-level coupling (top: CCV_total_, middle: CCV_noise_) and motion index (bottom) across high-selectivity sessions (5,244 trials from 25 sessions).
(TIF)

S7 FigCommunication subspaces of S1 and M2 align with their local stimulus coding subspace in total correlations.**(A–C)** Average CCV_total_ across sessions in each time window and delay (A), showing significant interaction in both directions (B, S1 → M2: *p*_up_ = 4.1 × 10^−8^, *z*_up_ = 5.5 and *p*_down_ = 1.7 × 10^−6^, *z*_down_ = −4.9; C, M2 → S1: *p*_up_ = 0.0009, *z*_up_ = 3.3, *p*_down_ = 3.5 × 10^−5^, *z*_down_ = −4.1, Wilcoxon signed-rank tests). **(D–F)** Average magnitude of similarity between ρd and cd_S1_ in each time window across sessions showing feedforward (E, S1 → M2: *p*_up_ = 5.9 × 10^−8^, *z*_up_ = 5.4) and feedback (F, M2 → S1: *p*_up_ = 0.001, *z*_up_ = 3.3, *p*_down_ = 0.07, *z*_down_ = −1.8, Wilcoxon signed-rank tests) components. **(G–I)** Average magnitude of similarity between ρd and cd_M2_ with significant feedforward (H, S1 → M2: *p*_up_ = 3.6 × 10^−8^, *z*_up_ = 5.5, and *p*_down_ = 0.002, *z*_down_ = −3.1) and feedback components (I, M2 → S1: *p*_up_ = 0.0002, *z*_up_ = 3.7, *p*_down_ = 0.017, *z*_down_ = −2.4, Wilcoxon signed-rank tests). Average data are represented as mean ± SEM. White lines indicate delay intervals selected for feedforward (FF) and feedback (FB) components. Lavender dashed lines represent 95% confidence interval of the baseline and gray dotted lines represent the shuffled CCV_total_. Wilcoxon signed-rank was used to test the differences between average upper and lower CI crossing points within 200 ms from the air puff onset vs. baseline (*n* = 50 sessions).(TIF)

S8 Fig Requirement of early S1 activity for behavioral discriminability and early M2 selectivity. **(A)** Schematic illustration of optogenetic suppression of S1 (blue). **(B)** Top: Behavioral discriminability (*d*’) in trials without (OFF) and with (ON) laser stimulation during the foil control, right S1 and left S1 sessions. Each color represents an individual mouse (*n* = 4), and connected diamonds show the within-session laser effect of laser stimulation. Bottom: Within-session change in behavioral discriminability induced by laser stimulation (∆*d*′ = *d*′_ON_ − *d*′_OFF_). Brief suppression of the right S1 produced a significantly larger reduction in *d*′ than either the foil control or left S1 suppression (repeated-measures ANOVA: *p* = 0.015, *F* = 9.2, *df* = 2, Fisher’s LSD: Control vs. Right S1: *p* = 0.019, Left S1 vs. Right S1: *p* = 0.042). The data underlying this figure can be found in https://doi.org/10.5281/zenodo.23338822 as S8B.xlsx. **(C)** Left: Average time course of M2 selectivity index during trials without (OFF, black) and with (ON, blue) right S1 suppression (mean ± SEM, *n* = 147 neurons). Right: Selectivity index of individual M2 neurons exhibiting significant selectivity within the first 100 ms after air puff during OFF and ON trials. Brief right S1 suppression significantly reduced early M2 selectivity (linear mixed-effects model: *p* = 0.0017, *t* = −3.17, *df* = 292; 9 sessions from the same 4 mice shown in B). Each gray line represents one neuron. Boxplots represent the median and interquartile range (IQR); whiskers extend to the most extreme data points within 1.5 × IQR of the first and the third quartiles, and notches represent the 95% confidence intervals of the median. The data underlying this figure can be found in https://doi.org/10.5281/zenodo.23338822 as S8C.xlsx
(TIF)

S9 Fig No change in coupling direction and coding-communication alignment in S1 and M2 after controlling for single-unit isolation quality. **(A)** Average autocorrelogram of well-isolated single units (blue; ~80% of recorded units) and excluded units with high inter-spike interval (ISI) violation rate (red) in S1 (left) and M2 (right). Shaded areas represent mean ± SEM across units. **(B)** Analysis of CCV_noise_ using only well-isolated single units. Top: Average CCV_noise_, S1 similarity, and M2 similarity maps across sessions. Middle: Feedforward coupling (left, *p*_up_ = 6.8 × 10^−5^, *z*_up_ = 4.0), S1 similarity (middle, *p*_*up*_ = 2.2 × 10^−5^, *z*_*up*_ = 4.2), and M2 similarity (right, *p*_*up*_ = 0.002, *z*_*up*_ = 3.0) remained significant after excluding low-quality units. Bottom: Feedback coupling and similarity measures showed no significant upper confidence interval (CI) crossing points. **(C)** Same as (B) but CCV_total_. Top: Average CCV_total_, S1 similarity, and M2 similarity maps across sessions. Middle: Feedforward coupling (left, *p*_up_ = 4.5 × 10^−8^, *z*_up_ = 5.5), S1 similarity (middle, *p*_*up*_ = 2.8 × 10^−8^, *z*_*up*_ = 5.5), and M2 similarity (right, *p*_*up*_ = 1.4 × 10^−6^, *z*_*up*_ = 4.8) remained significant after excluding low-quality units. Bottom: Feedback coupling (left, *p*_up_ = 0.0014, *z*_up_ = 3.2), S1 similarity (middle, *p*_*up*_ = 3.0 × 10^−4^, *z*_*up*_ = 3.6), and M2 similarity (right, *p*_*up*_ = 1.2 × 10^−4^, *z*_*up*_ = 3.8) showed significance. Data are represented as mean ± SEM. White horizontal lines indicate the delay windows used to quantify feedforward (top) and feedback (bottom) components. Lavender dashed lines represent the 95% confidence interval of the baseline, and gray dotted lines represent shuffled CCV. Upper and lower CI crossing points within 200 ms after air-puff onset were compared with baseline using the Wilcoxon signed-rank test (*n* = 50 sessions).
(TIF)

S10 Fig Split-half cross-validation confirms consistent feedforward coupling and coding-communication alignment in S1 and M2. **(A)** Cross-validated average maps of CCV_noise_, S1 similarity, and M2 similarity across sessions. Communication and coding subspaces were computed from independent halves of trials within each session. **(B)** Feedforward CCV_noise_ (left, *p*_up_ = 0.006, *z*_up_ = 2.7), cross-validated S1 similarity (middle, *p*_*up*_ = 0.0033, *z*_*up*_ = 2.9), and cross-validated M2 similarity (right, *p*_*up*_ = 0.033, *z*_*up*_ = 2.1), demonstrating consistent feedforward coupling and coding-communication alignment. **(C)** Feedback CCV_noise_ (left), cross-validated S1 similarity (middle), and cross-validated M2 similarity (right), showing no significant increase, consistent with the non-cross-validated results. **(D)** Cross-validated, average maps of CCV_total_, S1 similarity, and M2 similarity across sessions, computed as in (A). **(E)** Feedforward CCV_total_ (left, *p*_up_ = 3.4 × 10^−7^, *z*_up_ = 5.1), cross-validated S1 similarity (middle, *p*_*up*_ = 5.8 × 10^−6^, *z*_*up*_ = 4.5), and cross-validated M2 similarity (right, *p*_*up*_ = 5.8 × 10^−6^, *z*_*up*_ = 4.5), confirming consistent feedforward coupling and coding-communication alignment. **(F)** Feedback CCV_total_ (left, *p*_up_ = 0.063, *z*_up_ = 1.9), cross-validated S1 similarity (middle, *p*_*up*_ = 0.03, *z*_*up*_ = 2.2) and cross-validated M2 similarity (right, *p*_*up*_ = 0.01, *z*_*up*_ = 2.5). Data are represented as mean ± SEM. White horizontal lines indicate the delay windows used to quantify feedforward (top) and feedback (bottom) components. Lavender dashed lines represent the 95% confidence interval of the baseline, and gray dotted lines represent shuffled CCV_total_. Upper and lower CI crossing points within 200 ms after air-puff onset were compared with baseline using the Wilcoxon signed-rank test (*n* = 50 sessions).
(TIF)

S11 Fig Cross-validated CCVs for total (CCV_total,_ left) and noise (CCV_noise,_ right) correlations, showing a consistent transient rise in feedforward coupling within the first 100 ms after air-puff onset. (TIF)

S12 Fig Consistency of cosine similarity sign between S1 and M2. **(A)** Two example sessions showing CCV_noise_ (1st column), signed S1 similarity (2nd), signed M2 similarity (3rd), and the similarity product (4th) for each time bin. **(B)** The average similarity product across sessions revealed a strong, transient alignment between coding and communication subspaces selectively in the feedforward (FF) direction, consistent with the results obtained using absolute cosine similarities. Left: Average similarity product across 50 sessions for each time bin. Middle: Feedforward similarity product, showing a significant increase within the first 100 ms after stimulus onset (*p*_*up*_ = 5.9 × 10^−7^, *z*_*up*_ = 5.0, upper confidence interval (CI) crossing points compared with baseline, Wilcoxon signed-rank test). Right: Feedback (FB) similarity product, showing no significant change (*p*_down_ = 0.18, *z*_down_ = −1.3, lower CI crossing points compared with baseline, Wilcoxon signed-rank test).
(TIF)

S13 FigLow consistency of coding directions over trial time.**(A and B)** Consistency (Pearson’s correlations) of cd calculated in each 100-ms time bin relative to the air puff onset, in S1 and M2, respectively. Arrows point to consistency values relative to the cd used to compute the similarity (0–0.1s). The late cd (1–1.1 s) is chosen for comparison in C–F. **(C and D)** Similarity calculation across sessions for feedforward (FF) components in total (C, S1_cd_ vs. S1_late cd_: *p* = 9.4 × 10^−5^, *z* = 3.9, M2_cd_ vs. M2_late cd_: *p* = 1.0 × 10^−7^, *z* = 5.3) and noise (D, S1_cd_ vs. S1_late cd_: *p* = 0.0004, *z* = 3.6, M2_cd_ vs. M2_late cd_: *p* = 1.1 × 10^−7^, *z* = 5.3, Wilcoxon signed-rank tests, *n* = 50 sessions) interactions. **(E and F)** Similarity calculation across sessions for feedback (FB) components in total (E, S1_cd_ vs. S1_late cd_: *p* = 0.16, *z* = 1.4, M2_cd_ vs. M2_late cd_: *p* = 0.15, *z* = 1.4) and noise (F, S1_cd_ vs. S1_late cd_: *p* = 0.88, *z* = 0.1, M2_cd_ vs. M2_late cd_: *p* = 0.68, *z* = 0.4, Wilcoxon signed-rank tests, n = 50 sessions) correlations. Dark green lines and circles represent sessions. Light green lines are the median of the data and blue boxes show interquartile ranges. The data underlying C–F can be found in https://doi.org/10.5281/zenodo.23338822 as S13CDEF.xlsx(TIF)

S14 Fig Total correlations predominantly show a feedforward bias, but a delayed feedback alignment with selectivity emerges in the late activity of both regions. **(A)** Baseline-corrected CCV_total_ profile (top), S1 similarity (middle) and M2 similarity (bottom) averaged across sessions. **(B)** Early (left) and late (right) CCV_total_ (top), S1 similarity (middle) and M2 similarity (bottom) averaged (±SEM) across sessions within the first and the second 50 ms, respectively, for each delay. **(C)** Early (left) vs. late (right) delay of the peak CCV_total_ (top), S1 similarity (middle), and M2 similarity (bottom) for each session (CCV_total_: *p* = 0.875, *z* = 0.16, S1: *p* = 0.018, *z* = 2.4, M2: *p* = 0.002, *z* = 3.1, Wilcoxon signed-rank tests). **(D)** Total (top) and noise (bottom) interaction maps during inter-trial intervals (up to 200 ms before air puff) reveal a feedforward bias. **(E)** Average (±SEM) of CCV_total_ (top) and CCV_noise_ (bottom) during ITI for each delay across sessions. **(F)** Delay of the peaks in CCV_total_ (top) and CCV_noise_ (bottom) showing feedforward but larger values compared to post-stimulus CCV (C, top). **(G)** Delay of the peaks as a function of population selectivity for CCV_total_ (top) and CCV_noise_ (bottom). Each circle represents a session (n = 50, 18 mice).
(TIF)

S15 Fig Subsequent canonical dimensions display a feedforward bias in total correlations. **(A)** Average (±SEM) of CCV_total_ (top row), S1 similarity (middle row), and M2 similarity (bottom row) for each delay across canonical dimensions (columns). **(B)** Peak magnitude of CCV_total_ (top), S1 similarity (middle), and M2 similarity (bottom) across 4 canonical dimensions. **(C)** Delay of the CCV_total_ (top), S1 (middle), and M2 (bottom) similarity across canonical dimensions. Each gray line represents one session. Boxplots represent the median (green) and interquartile range (IQR, blue) of the data in each dimension and the whiskers extend to the last data point not considered an outlier. Red plus signs represent outliers (1.5 times IQR below and above the first and third quartiles, respectively). Notches represent 95% confidence intervals of medians. The data underlying B and C can be found in https://doi.org/10.5281/zenodo.23338822 as S15BC.xlsx
(TIF)

S16 Fig Consistently weak delay-period coupling across delay intervals, canonical dimensions and correlation types (total and noise), with no alignment between choice-coding and communication subspaces in either S1 or M2. Top three rows: CCV_total_ (top), S1 similarity (middle), and M2 similarity (bottom). Columns 1–4: Average values (±SEM) for the 1st through 4th canonical dimensions. Column 5: Peak magnitude across 4 canonical dimensions. Column 6: Peak delay across canonical dimensions. Gray lines represent individual sessions. Boxplots indicate the median (green line) and interquartile range (IQR, blue box); whiskers extend to the most extreme data points within 1.5 × IQR of the first and third quartiles, red crosses denote outliers, and notches represent the 95% confidence interval of the median. Bottom three rows: Same analyses as above, shown for CCV_noise_. The data underlying boxplots (right two columns) can be found in https://doi.org/10.5281/zenodo.23338822 as S16_BoxPlots.xlsx
(TIF)

S17 Fig Higher temporal resolution (1 ms) and jitter correction revealed short-delay coupling in the feedforward direction. **(A)** Total canonical correlation values (CCVs) were computed using 1 ms spike time binning in 50 ms windows with a 1 ms step size. **(B)** Average CCV within 50 ms of air puff shows a clear peak at a positive delay. **(C)** Distribution of the peak magnitudes of each session’s CCV across delays. **(D)** Delay of the peaks as a function of S1 population selectivity. **(E)** Average (±SEM) of raw (top row) and jitter-corrected (bottom row) CCVs as a function of canonical dimensions (columns) across 25 high-selectivity sessions within 50 ms of air puff. **(F)** Magnitude of peaks in raw (top), and jitter-corrected (bottom) CCVs across dimensions. **(G)** Absolute delay of the peaks in raw (top) and jitter-corrected (bottom) CCVs. **(H)** Average of raw (top row) and jitter-corrected (bottom row) CCVs as a function of canonical dimensions (columns) across 25 low-selectivity sessions within 50 ms of air puff. **(I)** Magnitude of peaks in raw (top), and jitter-corrected (bottom) CCVs in low-selectivity sessions. **(J)** Absolute delay of the peaks in raw (top) and jitter-corrected (bottom) CCVs in low-selectivity sessions. Boxplots represent the median (green) and interquartile range (IQR, blue) of the data in each dimension and the whiskers extend to the last data point not considered an outlier. Red plus signs represent outliers (1.5 times IQR below and above the first and the third quartiles, respectively). Notches represent 95% confidence intervals of medians. The data underlying F, G, I and J can be found in https://doi.org/10.5281/zenodo.23338822 as S17FGIJ.xlsx
(TIF)

S18 Fig Coupling delay estimation using time-shifted CCA in go vs. no-go trials. **(A-D)** Total canonical correlation values (CCV_total_) computed using go trials only (5-ms spike bins, 50-ms analysis window, 10-ms step size). **(A)** Average CCV map across sessions, **(B)** Average CCV as a function of delay. **(C)** Peak CCV magnitude versus peak delay of CCV for individual sessions. **(D)** Peak delay versus the fraction of selective S1 neurons (Frac. Sel. S1), showing a median peak delay of +5 ms (*p* = 0.0006, *z* = 3.43, Wilcoxon signed-rank test, *n* = 50 sessions). **(E-H)** CCV_total_ computed using no-go trials only. **(E)** Average CCV map across sessions. **(F)** Average CCV as a function of delay **(G)** Peak CCV magnitude versus peak delay for individual sessions. **(H)** Peak delay versus the fraction of selective S1 neurons (Frac. Sel. S1), showing no significant delay bias (median peak delay = 0 ms; *p* = 0.17*, z* = 1.37, *n* = 50 sessions). **(I, J)** Raser plots (black dots) and firing rates (red) of two simulated populations (70 neurons each) in an example trial. Population 1 (I) drove population 2 (J) within a predefined 50-ms coupling window with a ground-truth delay of 10 ms. **(K)** CCV_noise_ computed for simulated populations with ground-truth delays ranging from −10 ms (population 1 lagging population 2) to +10 ms (population 1 leading population 2). Time-shifted CCA accurately recovered the imposed coupling delays across the full range of simulated conditions.
(TIF)

S19 Fig Robustness of CCA-based coupling delay estimation across a range of parameters. **(A)** Stability of CCA-based peak delay estimation in simulated populations with a ground-truth delay of 4 ms across a range of analysis parameters: spike bin size (b), analysis window size (w), step size (s), and jitter window size (jw), all expressed in milliseconds. The top, middle, and bottom panels show the estimated peak delay (green circles) for parameters combinations using 1-, 5-, and 10-ms spike bin sizes, respectively. **(B)** Stability of CCA-based peak delay estimation in an example recording session across the same range of analysis parameters. The format is the same as (A).
(TIF)

S20 Fig Stability of CCA-based coupling delay estimation across the parameters for all recording sessions. **(A)** Distribution of peak delays for uncorrected CCV_total_ across the 50 recording sessions over a range of analysis parameters (b, spike bin size; w, analysis window size; s, step size; jw, jitter window size; all in milliseconds) using a 1-ms spike bin size. Green dots represent individual sessions and demonstrate consistent delay estimates across parameter combinations. **(B)** Distribution of peak delays for jitter-corrected CCV_total_ across the same parameter combinations using a 1-ms spike bin size, showing that the estimated delays are largely insensitive to the choice of jitter window. **(C)** Top: Probability density functions (pdfs) of estimated peak delays within ±10 ms for uncorrected (left, average = +1.7 ms, significantly larger than zero, *p* = 0.004, *n* = 50) and jitter-corrected (right, average = +1.1 ms, *p* = 0.021, *n* = 50, one-sided mouse-level sign-flip permutation tests) CCVs using a 1-ms spike bin size across parameter combinations. Bottom: Corresponding distributions obtained with a 5-ms spike bin size, showing consistent but more discretized peak-delay estimates. **(D)** Distribution of estimated peak delays in high- and low-selectivity sessions using a 1-ms spike bin size across parameter combinations. Top: Uncorrected CCVs (high-selectivity: average = +3.1 ms, *p* = 0, *n* = 25; low-selectivity: average = +0.26 ms, *p* = 0.38, *n* = 25). Bottom: Jitter-corrected CCVs (high-selectivity: average = +2.0 ms, *p* = 0.010, *n* = 25; low-selectivity: average = +0.25 ms, *p* = 0.35, *n* = 25, one-sided mouse-level sign-flip permutation tests). Left and right panels correspond to high- and low-selectivity sessions, respectively.
(TIF)

S21 Fig Pairwise cross-correlation analysis recapitulates the dominance of feedforward coupling between S1 and M2. **(A)** Left: Raw (uncorrected), z-scored pairwise cross-correlograms (CCGs; example session) computed within the first 100 ms after air puff. Many unit pairs exhibited peaks at short positive delays (red box, S1 → M2), consistent with fast, feedforward coupling. Right: Time- and unit-shuffled CCGs from the same session. Unit pairs are sorted by their peak delay. **(B)** Same as (A) but for jitter-corrected CCGs. **(C)** Distribution of the peak delays of raw CCGs across all unit pairs from high-selectivity sessions showing a peak at short positive delay (average = +1.5 ms, *p* = 0.002, average shuffled peak delay = −0.07 ms, *p* = 0.8, n = 25, one-sided mouse-level sign-flip permutation test on data within ±10ms). **(D)** Same as (C) but for jitter-corrected CCGs (average = +1.0 ms, *p* = 0.003, average shuffled peak delay = +0.05 ms, *p* = 0.1, *n* = 25). **(E, F)** Raw (E) and jitter-corrected (F) CCGs of unit pairs with the highest peak magnitudes (top 5%). Most pairs exhibited short positive delays consistent with the fast feedforward S1 → M2 coupling revealed by CCA. E: average raw peak delay = +2.2 ms, *p* = 0.002, shuffled data: −0.14 ms, *p* = 0.84 and F: average jitter-corrected peak delay = +1.8 ms, *p* = 0.002, shuffled data: −0.009 ms, *p* = 0.47, n = 25 high-selectivity sessions, one-sided mouse-level sign-flip permutation tests within ±10 ms).
(TIF)

S22 Fig Generalized linear modeling of S1–M2 interactions confirms the predominance of feedforward coupling identified by CCA. **(A)** Linear model (LM) applied to simulated neuronal populations accurately recovered the ground-truth interaction delays of −4 ms (neurons 1–20), 0 ms (neurons 21–40), and 4 ms (neurons 41–60). **(B)** LM applied to an example recording session within the first 100 ms after air puff onset. Most M2 neurons exhibited peak coupling weights at positive delays (S1 leading M2), consistent with feedforward interactions. **(C)** Distribution of peak interaction delays across sessions as a function of S1 selectivity recapitulated the peak delay intervals observed with CCA (S17D Fig). **(D)** Distribution of peak interaction delays across target neurons, estimated by the LM in high- and low-selectivity sessions (*p* = 8.3 × 10^−9^, *t* = 5.8, linear mixed-effects model with session-level selectivity as a fixed effect, *n* = 1,953 neurons with at least one non-zero coupling weight across delays). **(E)** A Poisson generalized linear model (GLM) yielded similar results, with peak interaction delays remaining predominantly positive and varying systematically with S1 selectivity (*p* = 6.6 × 10^−6^, *t* = 4.5, linear mixed-effects model, *n* = 2,917 neurons with at least one non-zero coupling weight across delays).
(TIF)

S23 Fig Unit isolation quality does not affect CCA-based delay estimation. **(A)** Average autocorrelograms of well-isolated single units (blue, ~80% of recorded units) and excluded units with high inter-spike interval (ISI) violation rates (red) in S1 (top) and M2 (bottom). Shaded areas represent mean ± SEM across units. **(B)** Average CCV_total_ map across sessions computed using only well-isolated single units (1-ms spike bins, 50-ms analysis windows, 50-ms step size). **(C)** Average CCV_total_ as a function of delay within the first 50 ms after air puff onset, showing a clear peak at positive delays. **(D)** Distribution of peak CCV_total_ magnitudes as a function of delay for individual sessions. **(E)** Peak delay as a function of S1 population selectivity. **(F–H)** CCV_total_ results for high-selectivity sessions. (F) Average raw (top) and jitter-corrected (bottom) CCVs across canonical dimensions within the first 50 ms after air-puff onset (mean ± SEM). (G) Peak CCV magnitude across canonical dimensions. (H) Absolute peak delay across canonical dimensions. **(I–K)** Same analyses as in F–H for low-selectivity sessions. Boxplots represent the median (green line) and interquartile range (IQR, blue box). Whiskers extend to the most extreme data points within 1.5 × IQR of the first and third quartiles, red crosses denote outliers, and notches represent the 95% confidence interval of the median. The data underlying G, H, J and K can be found in https://doi.org/10.5281/zenodo.23338822 as S23GHJK.xlsx
(TIF)

## Abbreviations

AUC: Area under ROC curve
CCA: canonical correlation analysis
CCG: cross-correlogram
CCV: canonical correlation value
CI: confidence interval
CR: correct rejection
FA: false alarm
GLM: generalized linear model
gmFR: geometric mean firing rate
IQR: interquartile range
ISI: inter-spike interval
LM: linear model
NACLAR: National Advisory Committee for Laboratory Animal Research
pdf: Probability density function
ROC: Receiver Operating Characteristic
SEM: standard error of the mean
SI: selectivity index

## References

[1] Sompolinsky H. Computational neuroscience: beyond the local circuit. Curr Opin Neurobiol. 2014;25:xiii–xviii. doi: 10.1016/j.conb.2014.02.002 24602868

[2] Harris KD, Mrsic-Flogel TD. Cortical connectivity and sensory coding. Nature. 2013;503(7474):51–8. doi: 10.1038/nature12654 24201278

[3] Niell CM, Scanziani M. How cortical circuits implement cortical computations: Mouse visual cortex as a model. Annual Review of Neuroscience. 2021;44:517–46. doi: 10.1146/annurev-neuro-102320-085825 PMC9925090 33914591

[4] Kamigaki T. Dissecting executive control circuits with neuron types. Neurosci Res. 2019;141:13–22. doi: 10.1016/j.neures.2018.07.004 30110598

[5] Kamigaki T. Prefrontal circuit organization for executive control. Neurosci Res. 2019;140:23–36. doi: 10.1016/j.neures.2018.08.017 30227166

[6] Miller EK, Lundqvist M, Bastos AM. Working Memory 2.0. Neuron. 2018;100:463–75. doi: 10.1016/j.neuron.2018.09.023 PMC8112390 30359609

[7] Liu D, Gu X, Zhu J, Zhang X, Han Z, Yan W, et al. Medial prefrontal activity during delay period contributes to learning of a working memory task. Science. 2014;346(6208):458–63. doi: 10.1126/science.1256573 25342800

[8] Zhu J, Cheng Q, Chen Y, Fan H, Han Z, Hou R, et al. Transient delay-period activity of agranular insular cortex controls working memory maintenance in learning novel tasks. Neuron. 2020;105(5):934-946.e5. doi: 10.1016/j.neuron.2019.12.008 32135091

[9] Kamigaki T, Dan Y. Delay activity of specific prefrontal interneuron subtypes modulates memory-guided behavior. Nat Neurosci. 2017;20(6):854–63. doi: 10.1038/nn.4554 28436982 PMC5554301

[10] Pinto L, Rajan K, DePasquale B, Thiberge SY, Tank DW, Brody CD. Task-dependent changes in the large-scale dynamics and necessity of cortical regions. Neuron. 2019;104(4):810-824.e9. doi: 10.1016/j.neuron.2019.08.025 31564591 PMC7036751

[11] Guo ZV, Li N, Huber D, Ophir E, Gutnisky D, Ting JT, et al. Flow of cortical activity underlying a tactile decision in mice. Neuron. 2014;81(1):179–94. doi: 10.1016/j.neuron.2013.10.020 24361077 PMC3984938

[12] Esmaeili V, Tamura K, Muscinelli SP, Modirshanechi A, Boscaglia M, Lee AB, et al. Rapid suppression and sustained activation of distinct cortical regions for a delayed sensory-triggered motor response. Neuron. 2021;109(13):2183-2201.e9. doi: 10.1016/j.neuron.2021.05.005 34077741 PMC8285666

[13] Huda R, Goard MJ, Pho GN, Sur M. Neural mechanisms of sensorimotor transformation and action selection. Eur J Neurosci. 2019;49(8):1055–60. doi: 10.1111/ejn.14069 30019473 PMC7350175

[14] Siegel M, Buschman TJ, Miller EK. Cortical information flow during flexible sensorimotor decisions. Science. 2015;348(6241):1352–5. doi: 10.1126/science.aab0551 26089513 PMC4721574

[15] Le Merre P, Esmaeili V, Charrière E, Galan K, Salin P-A, Petersen CCH, et al. Reward-based learning drives rapid sensory signals in medial prefrontal cortex and dorsal hippocampus necessary for goal-directed behavior. Neuron. 2018;97(1):83-91.e5. doi: 10.1016/j.neuron.2017.11.031 29249287 PMC5766832

[16] Crochet S, Lee S-H, Petersen CCH. Neural circuits for goal-directed sensorimotor transformations. Trends in Neurosciences. 2019;42:66–77. doi: 10.1016/j.tins.2018.08.011 30201180

[17] Diamond ME, Arabzadeh E. Whisker sensory system: From receptor to decision. Prog Neurobiol. 2013;103:28–40. doi: 10.1016/j.pneurobio.2012.05.013 22683381

[18] Adibi M. Whisker-mediated touch system in rodents: From neuron to behavior. Front Syst Neurosci. 2019;13:40. doi: 10.3389/fnsys.2019.00040 31496942 PMC6712080

[19] Chen JL, Carta S, Soldado-Magraner J, Schneider BL, Helmchen F. Behaviour-dependent recruitment of long-range projection neurons in somatosensory cortex. Nature. 2013;499(7458):336–40. doi: 10.1038/nature12236 23792559

[20] Ni J, Chen JL. Long-range cortical dynamics: a perspective from the mouse sensorimotor whisker system. Eur J Neurosci. 2017;46(8):2315–24. doi: 10.1111/ejn.13698 28921729

[21] Condylis C, Lowet E, Ni J, Bistrong K, Ouellette T, Josephs N. Context-dependent sensory processing across primary and secondary somatosensory cortex. Neuron. 2020;106:515-525.e5. doi: 10.1016/j.neuron.2020.02.004 PMC7210055 32164873

[22] Manita S, Suzuki T, Homma C, Matsumoto T, Odagawa M, Yamada K, et al. A top-down cortical circuit for accurate sensory perception. Neuron. 2015;86(5):1304–16. doi: 10.1016/j.neuron.2015.05.006 26004915

[23] Peters AJ, Marica A-M, Fabre JMJ, Harris KD, Carandini M. Visuomotor learning promotes visually evoked activity in the medial prefrontal cortex. Cell Rep. 2022;41(3):111487. doi: 10.1016/j.celrep.2022.111487 36261004 PMC9631115

[24] Arce-McShane FI, Ross CF, Takahashi K, Sessle BJ, Hatsopoulos NG. Primary motor and sensory cortical areas communicate via spatiotemporally coordinated networks at multiple frequencies. Proc Natl Acad Sci U S A. 2016;113(18):5083–8. doi: 10.1073/pnas.1600788113 27091982 PMC4983822

[25] Fries P. Rhythms for cognition: Communication through coherence. Neuron. 2015;88(1):220–35. doi: 10.1016/j.neuron.2015.09.034 26447583 PMC4605134

[26] Rogers BP, Morgan VL, Newton AT, Gore JC. Assessing functional connectivity in the human brain by fMRI. Magn Reson Imaging. 2007;25(10):1347–57. doi: 10.1016/j.mri.2007.03.007 17499467 PMC2169499

[27] Bastos AM, Schoffelen J-M. A tutorial review of functional connectivity analysis methods and their interpretational pitfalls. Front Syst Neurosci. 2016;9:175. doi: 10.3389/fnsys.2015.00175 26778976 PMC4705224

[28] Siegel M, Donner TH, Engel AK. Spectral fingerprints of large-scale neuronal interactions. Nat Rev Neurosci. 2012;13(2):121–34. doi: 10.1038/nrn3137 22233726

[29] Kumar A, Rotter S, Aertsen A. Spiking activity propagation in neuronal networks: Reconciling different perspectives on neural coding. Nat Rev Neurosci. 2010;11(9):615–27. doi: 10.1038/nrn2886 20725095

[30] Hahn G, Ponce-Alvarez A, Deco G, Aertsen A, Kumar A. Portraits of communication in neuronal networks. Nat Rev Neurosci. 2019;20(2):117–27. doi: 10.1038/s41583-018-0094-0 30552403

[31] Battaglia D, Witt A, Wolf F, Geisel T. Dynamic effective connectivity of inter-areal brain circuits. PLoS Comput Biol. 2012;8(3):e1002438. doi: 10.1371/journal.pcbi.1002438 22457614 PMC3310731

[32] Buzsáki G, Schomburg EW. What does gamma coherence tell us about inter-regional neural communication?. Nat Neurosci. 2015;18(4):484–9. doi: 10.1038/nn.3952 25706474 PMC4803441

[33] Gregoriou GG, Gotts SJ, Zhou H, Desimone R. High-frequency, long-range coupling between prefrontal and visual cortex during attention. Science. 2009;324(5931):1207–10. doi: 10.1126/science.1171402 19478185 PMC2849291

[34] Nácher V, Ledberg A, Deco G, Romo R. Coherent delta-band oscillations between cortical areas correlate with decision making. Proc Natl Acad Sci U S A. 2013;110(37):15085–90. doi: 10.1073/pnas.1314681110 23980180 PMC3773764

[35] Urai AE, Doiron B, Leifer AM, Churchland AK. Large-scale neural recordings call for new insights to link brain and behavior. Nat Neurosci. 2022;25(1):11–9. doi: 10.1038/s41593-021-00980-9 34980926

[36] Kim TH, Schnitzer MJ. Fluorescence imaging of large-scale neural ensemble dynamics. Cell. 2022;185(1):9–41. doi: 10.1016/j.cell.2021.12.007 34995519 PMC8849612

[37] Jun JJ, Steinmetz NA, Siegle JH, Denman DJ, Bauza M, Barbarits B, et al. Fully integrated silicon probes for high-density recording of neural activity. Nature. 2017;551(7679):232–6. doi: 10.1038/nature24636 29120427 PMC5955206

[38] Ebitz RB, Hayden BY. The population doctrine in cognitive neuroscience. Neuron. 2021;109(19):3055–68. doi: 10.1016/j.neuron.2021.07.011 34416170 PMC8725976

[39] Semedo JD, Gokcen E, Machens CK, Kohn A, Yu BM. Statistical methods for dissecting interactions between brain areas. Curr Opin Neurobiol. 2020;65:59–69. doi: 10.1016/j.conb.2020.09.009 33142111 PMC7935404

[40] Keeley SL, Zoltowski DM, Aoi MC, Pillow JW. Modeling statistical dependencies in multi-region spike train data. Curr Opin Neurobiol. 2020;65:194–202. doi: 10.1016/j.conb.2020.11.005 33334641 PMC7769979

[41] Panzeri S, Moroni M, Safaai H, Harvey CD. The structures and functions of correlations in neural population codes. Nat Rev Neurosci. 2022;23(9):551–67. doi: 10.1038/s41583-022-00606-4 35732917

[42] Quian Quiroga R, Panzeri S. Extracting information from neuronal populations: information theory and decoding approaches. Nat Rev Neurosci. 2009;10(3):173–85. doi: 10.1038/nrn2578 19229240

[43] Semedo JD, Jasper AI, Zandvakili A, Krishna A, Aschner A, Machens CK, et al. Feedforward and feedback interactions between visual cortical areas use different population activity patterns. Nat Commun. 2022;13(1):1099. doi: 10.1038/s41467-022-28552-w 35232956 PMC8888615

[44] Han S, Helmchen F. Coordinated multi-level adaptations across neocortical areas during task learning. Nat Commun. 2025;16(1):7719. doi: 10.1038/s41467-025-62949-7 40830364 PMC12365232

[45] Chen JL, Voigt FF, Javadzadeh M, Krueppel R, Helmchen F. Long-range population dynamics of anatomically defined neocortical networks. King AJ, editor. eLife. 2016;5: e14679. doi: 10.7554/eLife.14679 PMC4929001 27218452

[46] Kang B, Druckmann S. Approaches to inferring multi-regional interactions from simultaneous population recordings. Curr Opin Neurobiol. 2020;65:108–19. doi: 10.1016/j.conb.2020.10.004 PMC7853322 33227602

[47] Javadzadeh M, Hofer SB. Dynamic causal communication channels between neocortical areas. Neuron. 2022;110(15):2470-2483.e7. doi: 10.1016/j.neuron.2022.05.011 35690063 PMC9616801

[48] Semedo JD, Zandvakili A, Machens CK, Yu BM, Kohn A. Cortical areas interact through a communication subspace. Neuron. 2019;102(1):249-259.e4. doi: 10.1016/j.neuron.2019.01.026 30770252 PMC6449210

[49] Zingg B, Hintiryan H, Gou L, Song MY, Bay M, Bienkowski MS, et al. Neural networks of the mouse neocortex. Cell. 2014;156(5):1096–111. doi: 10.1016/j.cell.2014.02.023 24581503 PMC4169118

[50] Hooks BM, Mao T, Gutnisky DA, Yamawaki N, Svoboda K, Shepherd GMG. Organization of cortical and thalamic input to pyramidal neurons in mouse motor cortex. J Neurosci. 2013;33(2):748–60. doi: 10.1523/JNEUROSCI.4338-12.2013 23303952 PMC3710148

[51] Rocco-Donovan M, Ramos RL, Giraldo S, Brumberg JC. Characteristics of synaptic connections between rodent primary somatosensory and motor cortices. Somatosens Mot Res. 2011;28(3–4):63–72. doi: 10.3109/08990220.2011.606660 21879990

[52] Thompson E. Mouse Head. 2020. doi: 10.5281/zenodo.3925943

[53] Fazlali Z, Ranjbar-Slamloo Y, Adibi M, Arabzadeh E. Correlation between cortical state and locus coeruleus activity: Implications for sensory coding in rat barrel cortex. Front Neural Circuits. 2016;10:14. doi: 10.3389/fncir.2016.00014 27047339 PMC4805600

[54] Sachidhanandam S, Sreenivasan V, Kyriakatos A, Kremer Y, Petersen CCH. Membrane potential correlates of sensory perception in mouse barrel cortex. Nat Neurosci. 2013;16(11):1671–7. doi: 10.1038/nn.3532 24097038

[55] Ranjbar-Slamloo Y, Arabzadeh E. High-velocity stimulation evokes “dense” population response in layer 2/3 vibrissal cortex. J Neurophysiol. 2017;117(3):1218–28. doi: 10.1152/jn.00815.2016 28003414 PMC5349327

[56] Han S, Helmchen F. Behavior-relevant top-down cross-modal predictions in mouse neocortex. Nat Neurosci. 2024;27(2):298–308. doi: 10.1038/s41593-023-01534-x 38177341

[57] Kohn A, Smith MA. Stimulus dependence of neuronal correlation in primary visual cortex of the macaque. J Neurosci. 2005;25(14):3661–73. doi: 10.1523/JNEUROSCI.5106-04.2005 15814797 PMC6725370

[58] Cohen MR, Kohn A. Measuring and interpreting neuronal correlations. Nat Neurosci. 2011;14(7):811–9. doi: 10.1038/nn.2842 21709677 PMC3586814

[59] de la Rocha J, Doiron B, Shea-Brown E, Josić K, Reyes A. Correlation between neural spike trains increases with firing rate. Nature. 2007;448(7155):802–6. doi: 10.1038/nature06028 17700699

[60] Harris KD, Thiele A. Cortical state and attention. Nat Rev Neurosci. 2011;12(9):509–23. doi: 10.1038/nrn3084 21829219 PMC3324821

[61] Lee SH, Dan Y. Neuromodulation of brain states. Neuron. 2012;76:209–22. doi: 10.1016/j.neuron.2012.09.012 PMC3579548 23040816

[62] Zagha E, McCormick DA. Neural control of brain state. Curr Opin Neurobiol. 2014;29:178–86. doi: 10.1016/j.conb.2014.09.010 25310628 PMC4254046

[63] McCormick DA, Nestvogel DB, He BJ. Neuromodulation of brain state and behavior. Annu Rev Neurosci. 2020;43:391–415. doi: 10.1146/annurev-neuro-100219-105424 32250724 PMC12237593

[64] Schriver BJ, Bagdasarov S, Wang Q. Pupil-linked arousal modulates behavior in rats performing a whisker deflection direction discrimination task. J Neurophysiol. 2018;120(4):1655–70. doi: 10.1152/jn.00290.2018 29995602 PMC6230792

[65] Ranjbar-Slamloo Y, Arabzadeh E. Diverse tuning underlies sparse activity in layer 2/3 vibrissal cortex of awake mice. J Physiol. 2019;597(10):2803–17. doi: 10.1113/JP277506 30932197 PMC6826235

[66] Ahmed NY, Ranjbar-Slamloo Y, Al Abed AS, Gao L, Sontani Y, RCom-H’cheo-Gauthier A, et al. Er81 Transcription factor fine-tunes striatal cholinergic interneuron activity and drives habit formation. J Neurosci. 2021;41(20):4392–409. doi: 10.1523/JNEUROSCI.0967-20.2021 33849945 PMC8152610

[67] Wang BA, Veismann M, Banerjee A, Pleger B. Human orbitofrontal cortex signals decision outcomes to sensory cortex during behavioral adaptations. Nat Commun. 2023;14(1):3552. doi: 10.1038/s41467-023-38671-7 37322004 PMC10272188

[68] Li N, Daie K, Svoboda K, Druckmann S. Robust neuronal dynamics in premotor cortex during motor planning. Nature. 2016;532(7600):459–64. doi: 10.1038/nature17643 27074502 PMC5081260

[69] Allen WE, Chen MZ, Pichamoorthy N, Tien RH, Pachitariu M, Luo L, et al. Thirst regulates motivated behavior through modulation of brainwide neural population dynamics. Science. 2019;364(6437):253. doi: 10.1126/science.aav3932 30948440 PMC6711472

[70] Averbeck BB, Latham PE, Pouget A. Neural correlations, population coding and computation. Nat Rev Neurosci. 2006;7(5):358–66. doi: 10.1038/nrn1888 16760916

[71] Romo R, Hernández A, Zainos A, Salinas E. Correlated neuronal discharges that increase coding efficiency during perceptual discrimination. Neuron. 2003;38(4):649–57. doi: 10.1016/s0896-6273(03)00287-3 12765615

[72] Reich DS, Mechler F, Victor JD. Independent and redundant information in nearby cortical neurons. Science. 2001;294(5551):2566–8. doi: 10.1126/science.1065839 11752580

[73] Miyamoto D, Hirai D, Fung CCA, Inutsuka A, Odagawa M, Suzuki T, et al. Top-down cortical input during NREM sleep consolidates perceptual memory. Science. 2016;352(6291):1315–8. doi: 10.1126/science.aaf0902 27229145

[74] Petrof I, Viaene AN, Sherman SM. Properties of the primary somatosensory cortex projection to the primary motor cortex in the mouse. J Neurophysiol. 2015;113(7):2400–7. doi: 10.1152/jn.00949.2014 25632081 PMC4416606

[75] Ferezou I, Haiss F, Gentet LJ, Aronoff R, Weber B, Petersen CCH. Spatiotemporal dynamics of cortical sensorimotor integration in behaving mice. Neuron. 2007;56(5):907–23. doi: 10.1016/j.neuron.2007.10.007 18054865

[76] Smith MA, Kohn A. Spatial and temporal scales of neuronal correlation in primary visual cortex. J Neurosci. 2008;28(48):12591–603. doi: 10.1523/JNEUROSCI.2929-08.2008 19036953 PMC2656500

[77] Piña Novo D, Gao M, Fischer R, Richevaux L, Yu J, Barrett JM, et al. Cortical dynamics in hand/forelimb S1 and M1 evoked by brief photostimulation of the mouse’s hand. Elife. 2025;14:RP105112. doi: 10.7554/eLife.105112 40387088 PMC12088680

[78] Barthas F, Kwan AC. Secondary motor cortex: Where “Sensory” meets “Motor” in the rodent frontal cortex. Trends Neurosci. 2017;40(3):181–93. doi: 10.1016/j.tins.2016.11.006 28012708 PMC5339050

[79] Holey BE, Schneider DM. Sensation and expectation are embedded in mouse motor cortical activity. Cell Rep. 2024;43(7):114396. doi: 10.1016/j.celrep.2024.114396 38923464 PMC11304474

[80] Siniscalchi MJ, Phoumthipphavong V, Ali F, Lozano M, Kwan AC. Fast and slow transitions in frontal ensemble activity during flexible sensorimotor behavior. Nat Neurosci. 2016;19(9):1234–42. doi: 10.1038/nn.4342 27399844 PMC5003707

[81] Sul JH, Jo S, Lee D, Jung MW. Role of rodent secondary motor cortex in value-based action selection. Nat Neurosci. 2011;14(9):1202–8. doi: 10.1038/nn.2881 21841777 PMC3164897

[82] Ishizu K, Nishimoto S, Ueoka Y, Funamizu A. Localized and global representation of prior value, sensory evidence, and choice in male mouse cerebral cortex. Nat Commun. 2024;15(1):4071. doi: 10.1038/s41467-024-48338-6 38778078 PMC11111702

[83] Murakami M, Vicente MI, Costa GM, Mainen ZF. Neural antecedents of self-initiated actions in secondary motor cortex. Nat Neurosci. 2014;17(11):1574–82. doi: 10.1038/nn.3826 25262496

[84] Murakami M, Shteingart H, Loewenstein Y, Mainen ZF. Distinct sources of deterministic and stochastic components of action timing decisions in rodent frontal cortex. Neuron. 2017;94(4):908-919.e7. doi: 10.1016/j.neuron.2017.04.040 28521140

[85] Reep RL, Goodwin GS, Corwin JV. Topographic organization in the corticocortical connections of medial agranular cortex in rats. J Comp Neurol. 1990;294(2):262–80. doi: 10.1002/cne.902940210 2332532

[86] Van Eden CG, Lamme VAF, Uylings HBM. Heterotopic cortical afferents to the medial prefrontal cortex in the rat. A combined retrograde and anterograde tracer study. Eur J Neurosci. 1992;4(1):77–97. doi: 10.1111/j.1460-9568.1992.tb00111.x 12106444

[87] Chen JL, Margolis DJ, Stankov A, Sumanovski LT, Schneider BL, Helmchen F. Pathway-specific reorganization of projection neurons in somatosensory cortex during learning. Nat Neurosci. 2015;18(8):1101–8. doi: 10.1038/nn.4046 26098757

[88] Kwon SE, Yang H, Minamisawa G, O’Connor DH. Sensory and decision-related activity propagate in a cortical feedback loop during touch perception. Nat Neurosci. 2016;19(9):1243–9. doi: 10.1038/nn.4356 27437910 PMC5003632

[89] Yang H, Kwon SE, Severson KS, O’Connor DH. Origins of choice-related activity in mouse somatosensory cortex. Nat Neurosci. 2016;19(1):127–34. doi: 10.1038/nn.4183 26642088 PMC4696889

[90] Chang Y-T, Finkel EA, Xu D, O’Connor DH. Rule-based modulation of a sensorimotor transformation across cortical areas. Elife. 2024;12:RP92620. doi: 10.7554/eLife.92620 38842277 PMC11156468

[91] Levy WB, Baxter RA. Energy efficient neural codes. Neural Comput. 1996;8(3):531–43. doi: 10.1162/neco.1996.8.3.531 8868566

[92] Barlow HB. Trigger features, adaptation and economy of impulses. Information processing in the nervous system. Berlin, Heidelberg: Springer; 1969. p. 209–30. doi: 10.1007/978-3-642-87086-6_11

[93] Helmchen F, Gilad A, Chen JL. Neocortical dynamics during whisker-based sensory discrimination in head-restrained mice. Neuroscience. 2018;368:57–69. doi: 10.1016/j.neuroscience.2017.09.003 28919043 PMC5726900

[94] Ebbesen CL, Insanally MN, Kopec CD, Murakami M, Saiki A, Erlich JC. More than just a “Motor”: Recent surprises from the frontal cortex. J Neurosci. 2018;38(44):9402–13. doi: 10.1523/JNEUROSCI.1671-18.2018 30381432 PMC6209835

[95] Schneider DM, Mooney R. How movement modulates hearing. Annu Rev Neurosci. 2018;41:553–72. doi: 10.1146/annurev-neuro-072116-031215 29986164 PMC6201761

[96] Leinweber M, Ward DR, Sobczak JM, Attinger A, Keller GB. A sensorimotor circuit in mouse cortex for visual flow predictions. Neuron. 2017;95(6):1420-1432.e5. doi: 10.1016/j.neuron.2017.08.036 28910624

[97] Makino H, Ren C, Liu H, Kim AN, Kondapaneni N, Liu X, et al. Transformation of cortex-wide emergent properties during motor learning. Neuron. 2017;94(4):880-890.e8. doi: 10.1016/j.neuron.2017.04.015 28521138 PMC5502752

[98] Kim J-H, Ma D-H, Jung E, Choi I, Lee S-H. Gated feedforward inhibition in the frontal cortex releases goal-directed action. Nat Neurosci. 2021;24(10):1452–64. doi: 10.1038/s41593-021-00910-9 34413512

[99] Liu J, Liu D, Pu X, Zou K, Xie T, Li Y, et al. The secondary motor cortex-striatum circuit contributes to suppressing inappropriate responses in perceptual decision behavior. Neurosci Bull. 2023;39(10):1544–60. doi: 10.1007/s12264-023-01073-2 37253985 PMC10533474

[100] Hao MHZ, Kamigaki T. Dissociable cortical contributions to impulse control during waiting. Sci Adv. 2025;11(42):eadx4732. doi: 10.1126/sciadv.adx4732 41105776 PMC12533596

[101] Bellavance M-A, Takatoh J, Lu J, Demers M, Kleinfeld D, Wang F, et al. Parallel inhibitory and excitatory trigemino-facial feedback circuitry for reflexive vibrissa movement. Neuron. 2017;95(3):673-682.e4. doi: 10.1016/j.neuron.2017.06.045 28735746 PMC5845798

[102] Sreenivasan V, Esmaeili V, Kiritani T, Galan K, Crochet S, Petersen CCH. Movement initiation signals in mouse whisker motor cortex. Neuron. 2016;92(6):1368–82. doi: 10.1016/j.neuron.2016.12.001 28009277 PMC5196025

[103] Woolsey TA, Van der Loos H. The structural organization of layer IV in the somatosensory region (SI) of mouse cerebral cortex. The description of a cortical field composed of discrete cytoarchitectonic units. Brain Res. 1970;17(2):205–42. doi: 10.1016/0006-8993(70)90079-x 4904874

[104] Petersen CCH. The functional organization of the barrel cortex. Neuron. 2007;56(2):339–55. doi: 10.1016/j.neuron.2007.09.017 17964250

[105] Inagaki HK, Fontolan L, Romani S, Svoboda K. Discrete attractor dynamics underlies persistent activity in the frontal cortex. Nature. 2019;566(7743):212–7. doi: 10.1038/s41586-019-0919-7 30728503

[106] Finkelstein A, Fontolan L, Economo MN, Li N, Romani S, Svoboda K. Attractor dynamics gate cortical information flow during decision-making. Nat Neurosci. 2021;24(6):843–50. doi: 10.1038/s41593-021-00840-6 33875892

[107] Inagaki HK, Chen S, Daie K, Finkelstein A, Fontolan L, Romani S, et al. Neural algorithms and circuits for motor planning. Annu Rev Neurosci. 2022;45:249–71. doi: 10.1146/annurev-neuro-092021-121730 35316610

[108] Veuthey TL, Derosier K, Kondapavulur S, Ganguly K. Single-trial cross-area neural population dynamics during long-term skill learning. Nat Commun. 2020;11(1):4057. doi: 10.1038/s41467-020-17902-1 32792523 PMC7426952

[109] MacDowell CJ, Libby A, Jahn CI, Tafazoli S, Ardalan A, Buschman TJ. Multiplexed subspaces route neural activity across brain-wide networks. Nat Commun. 2025;16(1):3359. doi: 10.1038/s41467-025-58698-2 40204762 PMC11982558

[110] Chong HR, Ranjbar-Slamloo Y, Ho MZH, Ouyang X, Kamigaki T. Functional alterations of the prefrontal circuit underlying cognitive aging in mice. Nat Commun. 2023;14(1):7254. doi: 10.1038/s41467-023-43142-0 37945561 PMC10636129

[111] Ranjbar-Slamloo Y, Chong HR, Kamigaki T. Aging disrupts the link between network centrality and functional properties of prefrontal neurons during memory-guided behavior. Commun Biol. 2025;8(1):62. doi: 10.1038/s42003-025-07498-x 39820515 PMC11739477

[112] Hilgen G, Sorbaro M, Pirmoradian S, Muthmann J-O, Kepiro IE, Ullo S, et al. Unsupervised spike sorting for large-scale, high-density multielectrode arrays. Cell Rep. 2017;18(10):2521–32. doi: 10.1016/j.celrep.2017.02.038 28273464

[113] Buccino AP, Hurwitz CL, Garcia S, Magland J, Siegle JH, Hurwitz R, et al. SpikeInterface, a unified framework for spike sorting. Elife. 2020;9:e61834. doi: 10.7554/eLife.61834 33170122 PMC7704107

[114] Zhuang X, Yang Z, Cordes D. A technical review of canonical correlation analysis for neuroscience applications. Hum Brain Mapp. 2020;41(13):3807–33. doi: 10.1002/hbm.25090 32592530 PMC7416047

[115] Smith MA, Kohn A. Spatial and temporal scales of neuronal correlation in primary visual cortex. J Neurosci. 2008;28(48):12591–603. doi: 10.1523/JNEUROSCI.2929-08.2008 19036953 PMC2656500

[116] Trepka EB, Zhu S, Xia R, Chen X, Moore T. Functional interactions among neurons within single columns of macaque V1. Elife. 2022;11:e79322. doi: 10.7554/eLife.79322 36321687 PMC9662816

[117] Harrison MT, Geman S. A rate and history-preserving resampling algorithm for neural spike trains. Neural Comput. 2009;21(5):1244–58. doi: 10.1162/neco.2008.03-08-730 19018703 PMC3065177

[118] Allen reference atlas – mouse brain. Available from: https://atlas.brain-map.org

